# Hypoxia-driven microRNA-27b underlies pathologic cardiac endoreplication in heart disease

**DOI:** 10.1038/s41392-026-02656-x

**Published:** 2026-05-14

**Authors:** Peter Mirtschink, Ting Yuan, Corinne Bischof, Minh Duc Pham, Chaonan Zhu, Akshay Ware, Yijie Mao, Meiqian Wu, Eva-Maria Rogg, Katharina Bottermann, Suam Gonzalez-Gonoggia, Corinne Berthonneche, Bettina Gercken, Eman Hagag, Katrin Strassburger, Samuel Sossalla, Sebastian N. Stehr, Wesley Abplanalp, Nicola Zamboni, Fabio Martelli, Thierry Pedrazzini, Markus Stoffel, Stefanie Dimmeler, Jaya Krishnan

**Affiliations:** 1 Institute for Clinical Chemistry and Laboratory Medicine, University Hospital and Faculty of Medicine, Technische Universität Dresden, Dresden, Germany; 2 Institute of Cardiovascular Regeneration, Centre for Molecular Medicine, Goethe-University Frankfurt, Frankfurt am Main, Germany; 3 Department of Medicine III, Division of Cardiology/Nephrology/Angiology, Goethe-University Frankfurt, Frankfurt am Main, Germany; 4 DZHK (German Centre for Cardiovascular Research), partner site RheinMain, Frankfurt am Main, Germany; 5 Cardiopulmonary Institute, Frankfurt, Germany; 6 MRC Clinical Sciences Centre, Imperial College London, London, UK; 7 Genome Biologics, Frankfurt am Main, Germany; 8 Cardiovascular Assessment Facility, University of Lausanne and CHUV, Lausanne, Switzerland; 9 Department of Internal Medicine II, University Medical Center Regensburg, Regensburg, Germany; 10 Klinik für Kardiologie und Pneumologie, Georg-August-Universität Goettingen and DZHK (German Centre for Cardiovascular Research), Goettingen, Germany; 11 Department of Anesthesiology and Intensive Care Medicine, University of Leipzig Medical Center, Leipzig, Germany; 12 Institute of Molecular Systems Biology, ETH Zurich, Zurich, Switzerland; 13 Molecular Cardiology Laboratory, IRCCS Policlinico San Donato, San Donato Milanese, Milan, Italy; 14 School of Cardiovascular and Metabolic Medicine & Sciences, MRC/BHF Centre of Research Excellence in Advanced Cardiac Therapies, King’s College London, London, UK; 15 Institute of Molecular Health Sciences, ETH Zurich, Zurich, Switzerland

**Keywords:** Cardiovascular diseases, Target validation, Cardiology

## Abstract

Heart disease is characterized by stress-induced endoreplication preceding pathological cardiomyocyte overgrowth, yet the upstream regulatory mechanisms linking tissue hypoxia to aberrant cellular growth remain incompletely defined. Here, we identify cardiac hypoxia as a key determinant of endoreplication through activation of a hypoxia-inducible factor-1 alpha-microRNA regulatory axis that converges on mitochondrial energetic control. We show that stress-induced activation of hypoxia-inducible factor-1 alpha drives transcriptional induction of microRNA-27b-5p, which directly represses the ATP synthase subunit ATP5A1, resulting in impaired mitochondrial ATP synthesis and accumulation of intra-mitochondrial ADP. Elevated ADP serves as a rate-limiting cofactor for one-carbon metabolism, promoting formate production and de novo purine biosynthesis, thereby enabling pathological endoreplication and cardiomyocyte hypertrophic growth. Genetic gain- and loss-of-function studies targeting hypoxia-inducible factor-1 alpha, microRNA-27b, and ATP5A1 across multiple mouse models of cardiac stress, together with correlative analyses of human cardiac biopsies, establish a conserved and causal relationship between dysregulated mitochondrial energetics and pathological cardiac remodeling. Inhibition of microRNA-27b-5p attenuates established cardiac hypertrophy, improves cardiac function, and suppresses stress-induced multinucleation in vivo. Leveraging this mechanistic insight, we identify the clinically approved antifolate compound methotrexate as an effective inhibitor of stress-induced cardiac endoreplication and pathological hypertrophy in preclinical models. Collectively, these findings define a druggable hypoxia-driven metabolic pathway linking mitochondrial ATP homeostasis to pathological cardiomyocyte growth and suggest therapeutic opportunities for targeting maladaptive cardiac remodeling.

## Introduction

Left ventricular enlargement, occurring as a result of pathological cardiac overgrowth, is thought to serve as an adaptation to maintain cardiac output and function in response to hypertension, stenosis, infarction or congenital defects.^1^ With parallels to neoplastic expansion, cardiac hypertrophy increases tissue oxygen demand which is unmet due to a disconnect between neo-vascularization of ventricular tissue and accelerated cardiac growth, leading to establishment of hypoxic foci.^2,3^ Increased left ventricular wall stress and hypoxia stabilizes the transcription factor Hypoxia Inducible Factor (HIF)1α that plays key roles in human heart disease development and progression; and is sufficient and necessary to drive cardiac pathogenesis in mice.^4–8^

In previous work,^9^ we identified ATP5A1 (also known as ATP5F1A, encoding for the α-subunit of the ATP synthase catalytic F_1_ complex which forms together with the β-subunit, the catalytic domain through which the central stalk rotates to regenerate ATP from ADP and inorganic phosphate^10^) as downregulated in human hypertrophic cardiomyopathy (HCM) and aortic stenosis (AS) biopsies. Further, we demonstrated that repression of ATP5A1 serves to drive aberrant de novo purine biosynthesis leading to endomitosis and pathological cardiac growth. However, it remained unclear precisely how the implementation of constitutive ATP5A1 repression was achieved. Given the criticality of ATP Synthase function for healthy cell physiology and function, we were puzzled as to how (and why), cardiac mitochondria would respond to stressors by activating a mitochondria-driven pathologic cellular anabolic pathway at the expense of healthy cellular energetics. Although it is well-established that hypoxia suppresses mitochondrial function and that HIF1α plays a central role in repressing mitochondrial metabolism, the precise mechanisms by which HIF1α exerts these effect remain unclear.^11^

Here, we demonstrate that ATP5A1 repression is mediated by pathologic stress-induced activation of HIF1α and the downstream induction of miR-27b. Inhibition of ATP5A1 suppresses mitochondrial ATP synthase activity, leading to accumulation of intra-mitochondrial ADP that serves as a cofactor for the rate-limiting enzyme of the 1-carbon pathway regulating formate and purine biosynthesis.^12,13^ Consequently, MiR-27b inactivation in vitro and in mice attenuates pre-existing heart failure in response to surgery-mediated aortic stenosis [transverse aortic constriction (TAC)],^14^ while cardiac-specific miR-27b expression results in spontaneous cardiac overgrowth. In accord, the activation of this axis correlates with human pathological cardiac hypertrophy. Given the impact of this pathway across different cardiac indications and the high unmet need for effective therapies, we set out to identify Food and Drug Administration (FDA) and European Medicines Agency (EMA) approved compounds targeting the HIF1α-miR-27b-5p axis for potential repositioning. In screening for clinically approved compounds, we identified Methotrexate as an efficacious inhibitor of pathologic cardiac endoreplication and hypertrophy in small animal mouse studies. These findings reveal a therapeutically targetable stress-dependent pathway connecting deregulated mitochondrial ATP homeostasis with pathophysiological endoreplication in cardiomyocytes, resulting in multinucleation and pathological morphological growth.

## Results

### HIF1α regulates F_1_F_0_ ATP synthase activity via miR-27b

**Fig. 1:**
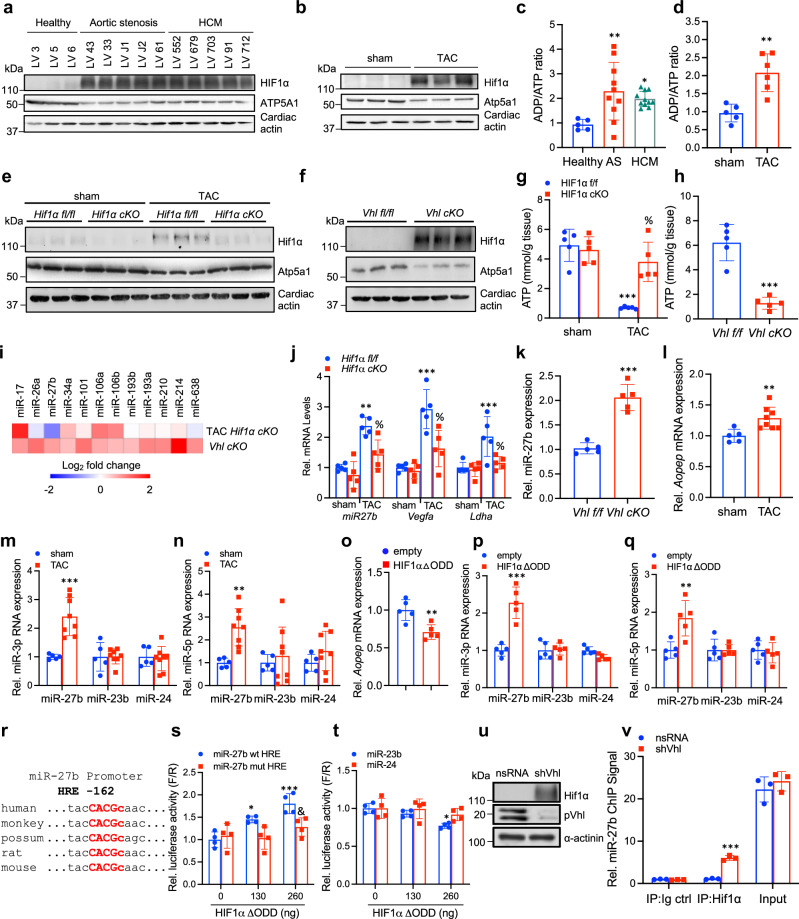
miR-27b is a direct HIF1α target. **a**, **b** Biopsies of left ventricles from patients with HCM and aortic stenosis and healthy controls (**a**) or sham- and TAC-operated mice (**b**) were assessed for denoted protein expression by immunoblotting. Loading is normalized to cardiac actin. **c** **d** ADP/ATP ratio in ventricular biopsies from HCM and aortic stenosis patients versus healthy controls (**c**), and in ventricular biopsies from mice subjected to TAC versus sham-operated animals (**d**). *n* = 4 for healthy controls; *n* = 10 for aortic stenosis and *n* = 11 for HCM in (**c**); *n* = 5 for sham and *n* = 6 for TAC in (**d**); shown is mean ± SD; *P < 0.05; **P < 0.01, one-way ANOVA followed by Dunnett’s post test (**c**) or two-tailed unpaired t-test (**d**). **e**, **f** Ventricular lysates of sham- or TAC-operated control (*Hif1α fl/fl*) and *Hif1α cKO* mice (**e**), or control (*Vhl fl/fl*) and *Vhl cKO* mice (**f**) were processed for immunoblotting with antibodies against Hif1α and Atp5a1. Loading is normalized to cardiac actin. **g**, **h** Quantification of ATP amount in left ventricular biopsies from control (*Hif1α fl/fl*) and ventricle-specific *Hif1α* conditional knockout (*Hif1α cKO*) mice subjected to sham or TAC surgery (**g**), and from ventricle-specific *Vhl* conditional knockout (*Vhl cKO*) and respective control (*Vhl fl/fl)* mice (**h**). *n* = 5 mice per group for (**g**) and (**h**); shown is mean ± SD; ***P < 0.0001 vs. sham *Hif1α fl/fl*, % *P* < 0.05 vs. TAC *Hif1α fl/fl* ; one-way ANOVA and Tukey’s post hoc test (**g**) and two-tailed unpaired t-test (**h**). **i** Expression profile heatmap of miRNAs containing putative HRE(s) in their promoter (*p* < 0.0001 between technical quadruplicates, and *p* < 0.05 of the biological triplicates with average signal intensity of 1-fold over background) from ventricular lysates of *Hif1α cKO* mice subjected to TAC surgery versus TAC-operated control animals, or *Vhl cKO* mice versus control animals. **j** Relative expression of *miR-27b*, *vascular endothelial growth factor* (*Vegfa*) and lactate dehydrogenase A (*Ldha)* mRNA in ventricles of control (*Hif1α fl/fl*) *and Hif1α cKO* mice subjected to sham or TAC surgery. Data is normalized to sham-operated controls (set as 1.0). *n* = 5 mice per group; shown is mean ± SD; ***P < 0.0001 vs. sham *Hif1α fl/fl*, % *P* < 0.01 vs. TAC *Hif1α fl/fl*; two-way ANOVA and Tukey’s post-hoc test. **k** Relative expression of miR-27b RNA in ventricles of control (*Vhl fl/fl*) and *Vhl cKO* mice. Data is normalized to control mice (set as 1.0). *n* = 5 mice per group; shown is mean ± SD; ***P < 0.001; two-tailed unpaired t-test. **l**–**n** Relative mRNA expression of *Aopep* (**l**), mature miR-27b-3p, miR-23b-3p and miR-24-3p (**m**), and miR27b-5p, miR23b-5p and miR24-5p (**m**) from ventricular lysates of wild-type C57BL/6 J mice subjected to sham or TAC surgery. Data is normalized to sham-operated control mice (set as 1.0). *n* = 5 for sham and *n* = 8 for TAC; data shown is mean ± SD; **P < 0.01; ***P < 0.001; two-tailed unpaired t-test. **o**–**q** Relative mRNA expression of *Aopep* (**o**), mature miR-27b-3p, miR-23b-3p and miR-24-3p (**p**), and miR-27b-5p, miR-23b-5p and miR-24-5p (**q**) in NRCs ectopically expressing an empty control vector or HIF1α. Data is normalized to NRCs transduced with an empty control vector (set as 1.0). *n* = 5 biological replicates per group; shown is mean ± SD; **P < 0.01, ***P < 0.001; two-tailed unpaired t-test. **r** Sequence of the human, monkey, possum, rat and mouse miR-27b promoter harboring a conserved HRE located 162 bp upstream of the precursor miR-27b transcription start site. HRE is shown in red, with the core HRE motif capitalized. **s** Co-transfection of wild type (wt) or HRE-mutated (mut) miR-27b promoters fused to luciferase with different doses of either an empty control vector or HIF1α ΔODD. Data is normalized to wild-type promoter transfected with empty control vector (set as 1.0). *n* = 4 biological replicates per group; data shown is mean ± SD; *P < 0.05; ***P < 0.001, & <0.01 vs. miR-27b wt HRE. Two-way ANOVA followed by Tukey’s post test. **t** Co-transfection of mir23b and mir24-1 promoters fused to luciferase with different doses of either an empty vector control or HIF1α ΔODD. Data is normalized to luciferase vector transfected with empty control vector (set as 1.0). *n* = 4 biological replicates per group; data shown is mean ± SD; *P < 0.05; one-way ANOVA followed by Tukey’s post test. **u** NRCs transduced with nsRNA or shVhl were assessed for Hif1α and Vhl protein levels by immunoblotting. Loading is normalized to cardiac actin. **v** NRCs were transduced with lentivirus expressing either a nsRNA or shRNA against Vhl (shVhl) and processed for chromatin immunoprecipitation with a HIF1α-specific antibody (IP: HIF1α) or with a control isotype-matched antibody (IgG control). Hif1α promoter binding was analyzed by qPCR. Data shown is relative to chromatin immunoprecipitation with Ig control antibody on nuclear lysates from NRCs expressing nsRNA (set as 1.0). *n* = 3 biological replicates per group; data shown is mean ± SD; ***P < 0.001; two-tailed unpaired t-test

We detected an inverse correlation between HIF1α and ATP5A1 protein levels across human and mouse cardiac biopsies of diseased left ventricles (Fig. 1a, b). Consistent with its established function,^9^ the reduction in ATP5A1 protein in human and mouse disease biopsies led to a concomitant decrease in ATP (Fig. 1c, d). Further, in utilizing conditional mouse models targeting HIF1α or the *von Hippel Lindau* protein (*Vhl*) (a negative regulator of HIF1α accumulation and function^6,7,15^), we aimed to confirm the relationship between Hif1α function and Atp5a1 expression. Cardiac ventricular-specific *Hif1α* conditional knockout (cKO) mice and *Hif1α* fl/fl littermate controls were subjected to TAC surgery, as a model for human aortic stenosis.^16^ While TAC surgery induced HIF1α activation and concomitant suppression of ATP5A1 expression in ventricular biopsies of control mice, mice deficient for Hif1α maintained ATP5A1 protein expression with abundance comparable to sham treated animals (Fig. 1e). In contrast, under gain-of-function settings wherein ventricular-specific Vhl inactivation serves to induce constitutive HIF1α accumulation and function, we observed prominent activation of Hif1α and suppression of Atp5a1 expression (Fig. 1f). Consistent with earlier findings, suppression of ATP5A1 expression in both settings led to a dramatic reduction in cellular ATP (Fig. 1g, h). Critically, decreased ATP abundance in the respective models correlated with reduced ejection fraction, indicative of cardiac systolic dysfunction (Supplementary Fig. 1a, b) Thus, our data reveals a causal role for HIF1α in regulating ATP5A1 protein expression, and by extension cellular ATP levels and cardiac function.

In assuming a transcriptional modulatory role for HIF1α in ATP5A1 regulation, we performed in silico analyses and expression studies^7^ to identify potential HIF1α-interaction sites at the ATP5A1 loci, or alternatively pinpoint direct HIF1α target genes capable of mediating the observed effects on *Atp5a1* mRNA expression and ATP abundance. Despite the prominent association between HIF1α function and ATP5A1 expression we repeatedly failed to identify such targets. This led us to explore HIF1α-regulated miRNAs as intermediaries in mediating these effects. We performed expression profiling of miRNAs from left ventricles of control and ventricular-specific *Hif1α* cKO mice subjected to TAC, and on ventricle-specific *Vhl* cKO mice. By comparing expression data within the respective groups and filtering for miRNAs containing cross-species conserved hypoxia response element (HRE) motifs,^17^ we identified several potential Hif1α-regulated miRNAs (Fig. 1i). A subset of these miRNAs have been previously linked to hypoxia and/or HIF1α dependence.^15,18–21^ Of these miRNAs, miR-27b was unique by its repression in *Hif1α* cKO mice subjected to TAC (compared to littermate control *Hif1α* fl/fl TAC mice) in line with the expression pattern of established HIF1α target genes, and its upregulation in ventricular-specific *Vhl* cKO mice (Fig. 1j, k), suggesting potential regulation by HIF1α.

MiR-27b is contained within the miR-23b-27b-24 cluster in intron 15 of the *aminopeptidase O* (*Aopep*) gene in humans and mice.^22^ To determine the stress- and Hif1α-dependence of miR-27b transcription, we assayed expression of *Aopep* and all miRNAs contained within the cluster in mice subjected to TAC, and in vitro in response to expression of a constitutively active HIF1α mutant lacking the oxygen dependent degradation domain (ODD) (referred to as HIF1α ΔODD)^23^ or by stimulation with the hypertrophy-inducing β-adrenergic receptor agonist Triiodothyronine (T3) (Fig. 1l–q and Supplementary Fig. 1c–f).^24^ Application of TAC in mice resulted in a slight induction of the host gene *Aopep* and unchanged expression of mature miR-23b-3p/5p and miR-24-3p/5p, whereas a ~2.5-fold induction of miR-27b-3p/5p was noted (Fig. 1l–n). In vitro ectopic HIF1α ΔODD expression led to downregulation of the host gene *Aopep* and unchanged levels of miR-23b-3p/5p and miR-24-3p/5p (Fig. 1o–q), while T3 stimulation induced *Aopep* expression but not that of miR-23b-3p/5p or miR-24-3p/5p (Supplementary Fig. 1d–f). In contrast, mature miR-27b-3p/5p was consistently upregulated in both in vitro models (Fig. 1o–q, Supplementary Fig. 1e, f). These results suggest that miR-27b-3p/5p is regulated independently of its host gene *Aopep* and its neighboring miRNAs in response to stress and HIF1α. To confirm miR-27b as a direct HIF1α target, we stimulated control non-silencing (ns) and shHif1α transduced neonatal rat cardiomyocytes (NRC) with T3 and analyzed the expression of miR-27b and established HIF1α target genes as controls (Supplementary Fig. 1g). In silico analysis for cross-species conserved HREs in the miR-27b promoter, corresponding to −1 kb upstream of the pre-miR-27b transcriptional start site (TSS), revealed a conserved HRE motif close to the TSS in humans and mice (Fig. 1r).^25^ To assess functionality of this HRE, miR-27b promoter-luciferase assays with the wild type and HRE-mutated promoter were performed. HIF1α ΔODD increased luciferase reporter expression only of the wild-type promoter but not in the HRE mutant promoter or in control cells, respectively (Fig. 1s). Promoters of the neighboring miRNAs miR-23b and miR-24 did not show increased promoter activity in the presence of ectopic expression of HIF1α ΔODD (Fig. 1t). Moreover, HIF1α specifically associated with the miR-27b promoter in native chromatin of NRC transduced with shVhl as revealed by Hif1α chromatin-immunoprecipitation (ChIP) from nuclear extracts (Fig. 1u, v). The efficiency of pVHL depletion by shVhl and consequent Hiflα accumulation was confirmed by immunoblotting (Fig. 1u). Taken together, these data define miR-27B as a bona fide HIF1α target miRNA.

**Fig. 2:**
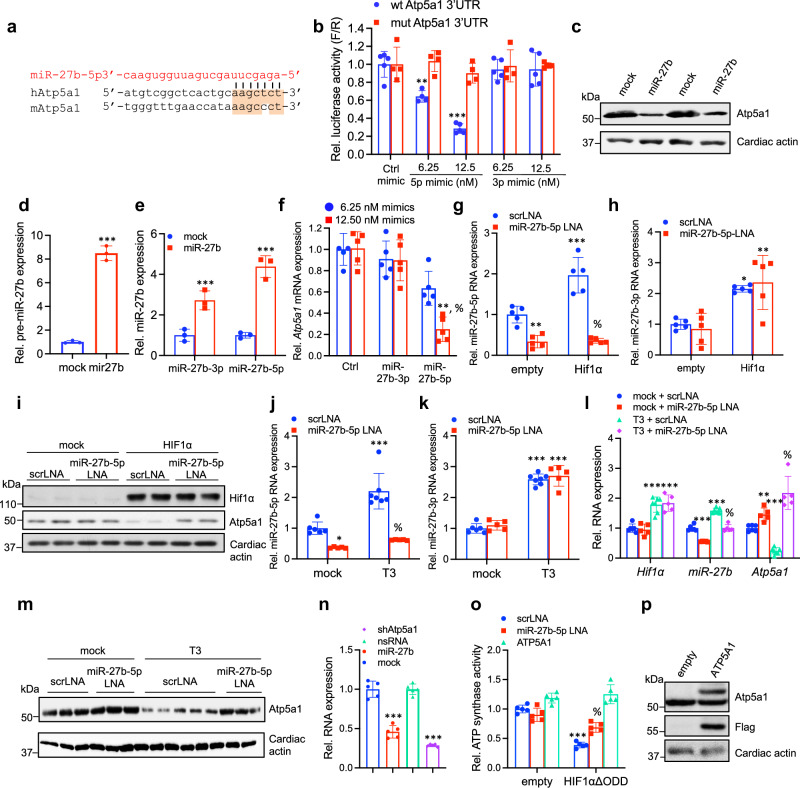
HIF1α regulates F_1_F_0_ ATP synthase activity via miR-27b. **a** Target gene recognition motif in the 3’UTR of human and mouse *Atp5a1*. The miR-27b-5p seed sequence and corresponding target region on Atp5a1 are indicated by alignment. **b** Co-transfection of wild type (wt) or miR-27b binding site-mutated (mut) Atp5a1 3’UTR fused to luciferase with different doses of control, miR-27b-3p or miR-27b-5p mimics. Data is normalized to wild-type promoter transfected with control mimics (set as 1.0) (*n* = 4 biological replicates per group; data shown is mean ± SD; **P < 0.01; ***P < 0.001; two-way ANOVA followed by Šidák’s post-test). **c** NRCs transduced with empty control vector or ectopic miR-27b were assessed for Atp5a1 protein levels by immunoblotting. Loading is normalized to cardiac actin. **d**, **e** Relative expression of miR-27b precursor (**d**) and mature miR-27b-3p and miR-27b-5p (**e**) in NRCs ectopically expressing an empty control vector or miR-27b. Data is normalized to NRCs transduced with an empty control vector (set as 1.0) (*n* = 3 biological replicates per group; shown is mean ± SD; ***P < 0.001; two-tailed unpaired t-test). **f** Relative expression level of *Atp5a1* mRNA from NRCs transfected with different concentrations of control, miR-27b-3p and miR-27b-5p mimics. Data is normalized to control mimics (set as 1.0) (*n* = 3 replicates per group; data shown is mean ± SD; **P < 0.01; % *P* < 0.01 compared to 6.25 mM miR-27b-5p mimics; Two-way ANOVA followed by Tukey’s post-test). **g**, **h** Relative expression of mature miR-27b-5p (**g**) and miR-27b-3p (**h**) in NRCs transduced with an empty vector control or HIF1α and treated with either scrLNA or miR-27b-5p LNAs. Data is normalized to NRCs transduced with an empty control vector (set as 1.0). (*n* = 5 biological replicates per group; shown is mean ± SD; *P < 0.05, **P < 0.01, ***P < 0.001 vs. empty scrLNA, % *P* < 0.05 vs. HIF1α + scrLNA (**g**); Two-way ANOVA followed by Tukey’s post-test). **i** Immunoblot for Hif1α and Atp5a1 expression in NRCs transduced with lentivirus expressing empty control vector or HIF1α and treated with either scrambled control (scrLNA) or miR27b-5p LNAs. Loading is normalized to cardiac actin. **j**, **k** Relative expression of mature miR-27b-5p (**j**) and miR-27b-3p (**k**) in NRCs treated with PBS (mock) or T3 in the presence of a scrLNA or miR-27b-5p LNA. Data is normalized to mock-treated cells (set as 1.0) (*n* = 5–7 biological replicates per group; shown is mean ± SD; *P < 0.05, ***P < 0.001, % *P* < 0.001 vs. T3 + scrLNA; Two-way ANOVA and Tukey’s post-test). **i** NRCs treated with PBS (mock) or T3 in the presence of scrLNA or miR-27b-5p LNA were assessed for *Hif1α*, *miR-27b* and *Atp5a1* RNA levels. Data is normalized to NRCs treated with scrLNA (set as 1.0). (*n* = 5–7 biological replicates per group; shown is mean ± SD; **P < 0.01, **P < 0.001 vs. mock scrLNA, % *P* < 0.001 vs. T3 + scrLNA; Two-way ANOVA and Tukey’s post-test). **m** NRCs treated as in (**l**) were assessed for Atp5a1 protein levels by immunoblotting. Loading is normalized to cardiac actin. **n**, **o** ATP synthase enzymatic activity in NRCs transduced and treated as indicated. Data is normalized to control sets (set as 1.0) (*n* = 5 biological replicates per group; results shown are the mean ± SD; ***P < 0.001 vs. mock/empty + scrLNA, % *P* < 0.001 vs. HIF1αΔΟΔΔ + scr LNA; two-tailed unpaired t-test (**n**) or Two-way ANOVA followed by Tukey’s post test (**o**). **p** NRCs transduced with empty control vector or ectopic ATP5A1 were assessed for Atp5a1 protein levels by immunoblotting. Loading is normalized to cardiac actin

ATP5A1 harbors a miR-27b-binding site in the 3’UTR, conserved between human and mice (Fig. 2a), which upon disruption by site-directed mutagenesis, results in de-repression of mir27b-mediated inhibition (Fig. 2b). In accord, ectopic miR-27b expression in NRC resulted in pronounced repression of Atp5a1 protein and mRNA (Fig. 2c–e). Despite upregulation of both miR-27b-3p and miR-27b-5p RNA by HIF1α, miR27b-5p was unique by its capacity to inhibit wildtype *Atp5a1* UTR-reporter expression (Fig. 2f). Treatment of cardiomyocytes with miR-27b-5p mimics led to the dose-dependent downregulation of *Atp5a1* mRNA but miR-27b-3p mimics did not (Fig. 2f). These observations were recapitulated with locked nucleic acid (LNA)-mediated miR-27b-5p inhibition in cardiomyocytes expressing ectopic HIF1α (Fig. 2g–i). LNAs specifically targeting miR-27b-5p led to de-repression of HIF1α induced ATP5A1 downregulation (Fig. 2g–i). Similar results were observed upon T3 stimulation where ATP5A1 repression was rescued upon simultaneous treatment with miR-27b-5p LNAs (Fig. 2j–m). Thus, miR-27b inhibition of ATP5a1 is mediated specifically by the miR-27b-5p species.

In order to determine if downregulation of ATP5A1 under these settings affected ATP synthase activity, we measured the ATP generating capacity of immunoprecipitated ATP synthase isolated from rat cardiomyocytes ectopically expressing miR-27b, or shAtp5a1 as positive control; or upon ectopic HIF1α expression in combination with miR-27b-5p LNAs (Fig. 2n, o). As noted, ectopic miR-27b and HIF1α expression led to significant repression of ATP synthase activity, with activity rescued upon parallel treatment with miR-27b-5p LNAs (Fig. 2n, o). ATP5A1 inactivation also mimicked the effects of ectopic miR-27b expression, while concomitant ATP5A1 overexpression with a flag-tagged construct (Fig. 2p) rescued the diminished ATP synthase activity upon ectopic HIF1α expression (Fig. 2o). Thus, ATP synthase activity is highly dependent on ATP5A1 expression levels.

### miR-27b inhibits ATP5A1 expression to drive cardiac hypertrophy and dysfunction

**Fig. 3:**
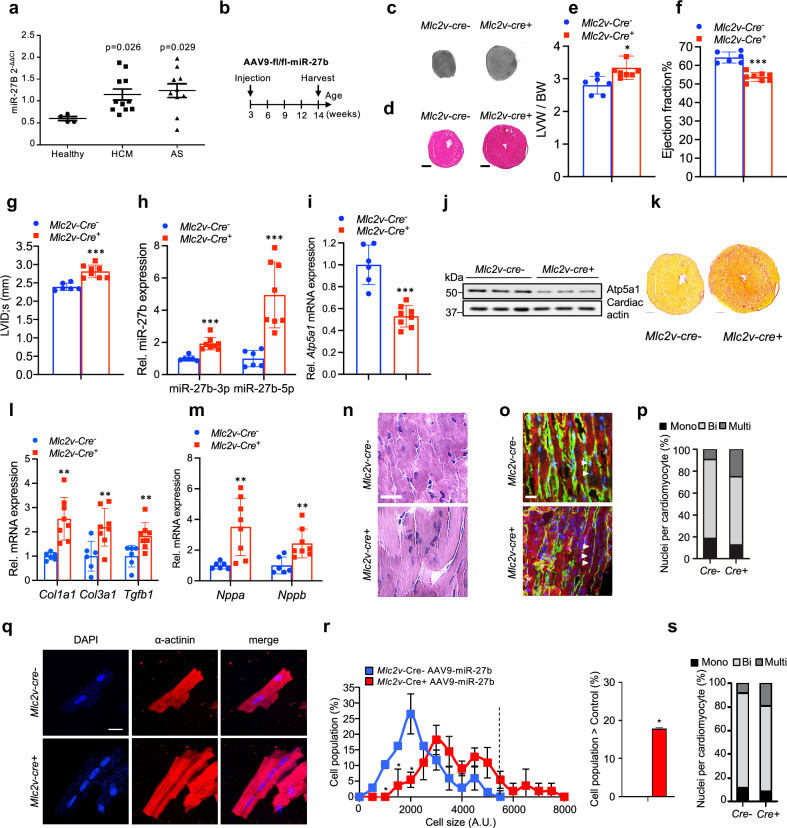
miR-27b controls cardiac ploidy and overgrowth. **a** Expression of miR-27B in patients with hypertrophic cardiomyopathy (HCM) or aortic stenosis (AS) (*n* = 4 for healthy control, *n* = 11 for HCM and *n* = 10 for AS; one-way ANOVA followed by Dunnett’s post test). **b** Schematic representation of the experimental timeline of *Mlc2v-cre* mice transduced with AAV9-loxP-STOPcassette-loxP-miR-27b. **c** Representative images of left ventricles of *Mlc2v-cre*^*+*^ and *Mlc2v-cre*^*-*^ mice injected with AAV9-loxP-STOPcassette-loxP-miR-27b viruses. **d** Representative images of H&E-stained histological sections of *Mlc2v-cre*^*+*^ and *Mlc2v-cre*^*-*^ mice transduced with AAV9-loxP-STOPcassette-loxP-miR-27b viruses. Scale bar is 1 mm. **e**–**g** LVW/BW (**e**), ejection fraction (**f**) and left ventricular internal diameter at systole (LVID;s) (**g**) of *Mlc2v-cre*^*+*^ and *Mlc2v-cre*^*-*^ mice injected with AAV9-loxP-STOPcassette-loxP-miR-27b viruses (*n* = 6 for *Mlc2v-cre*^*-*^ and *n* = 8 for *Mlc2v-cre*^*+*^ mice; results shown are the mean ± SD; *P < 0.05, ***P < 0.001; two-tailed unpaired t-test). **h** Relative expression of mature miR-27b-5p and miR-27b-3p in Mlc2v-cre+ and Mlc2v-cre^-^ mice injected with AAV9-loxP-STOPcassette-loxP-miR-27b viruses. *n* = 6 for Mlc2v-cre- and *n* = 8 for Mlc2v-cre+ mice; results shown are the mean ± SD; ***P < 0.001; two-tailed unpaired t-test. **i** Relative expression of *Atp5a1* mRNA in *Mlc2v-cre*^*+*^ and *Mlc2v-cre*^*-*^ mice transduced with AAV9-loxP-STOPcassette-loxP-miR-27b 11 weeks after AAV9 injection by qPCR. Data is normalized to *Mlc2v-cre*^*-*^ mice transduced with AAV9-loxP-STOPcassette-loxP-miR-27b (set as 1.0). *n* = 6 for *Mlc2v-cre*^*-*^ and *n* = 8 for *Mlc2v-cre*^*+*^ mice; shown is mean ± SD; ***P < 0.001; two-tailed unpaired t-test. **j** Immunoblot for Atp5a1 protein in ventricular lysates of *Mlc2v-cre*^*+*^ and *Mlc2v-cre*^*-*^ mice transduced with AAV9-loxP-STOPcassette-loxP-miR-27b viruses. Loading is normalized to cardiac actin. **k** Representative images of picrosirius red-stained histological sections of *Mlc2v-cre*^*+*^ and *Mlc2v-cre*^*-*^ mice transduced with AAV9-loxP-STOPcassette-loxP-miR-27b viruses. Scale bar is 500 μm. **l** Relative expression of *Col1a1*, *Col3a1* and *TGFb1* mRNA in left ventricular lysates of *Mlc2v-cre*^*+*^ and *Mlc2v-cre*^*-*^ mice transduced with AAV9-fl/fl-miR-27b viruses. Data is normalized to *Mlc2v-cre*^*-*^ mice (set as 1.0). *n* = 6 for *Mlc2v-cre*^*-*^ and *n* = 8 for *Mlc2v-cre*^*+*^ mice; results shown are the mean ± SD; *P < 0.01; two-tailed unpaired t-test. **m** Relative expression of *Nppa* and *Nppb* mRNA in *Mlc2v-cre*^*+*^ and *Mlc2v-cre*^*-*^ mice transduced with AAV9-loxP-STOPcassette-loxP-miR-27b viruses. *n* = 6 for Mlc2v-cre- and *n* = 8 for Mlc2v-cre+ mice; results shown are the mean ± SD; **P < 0.01; two-tailed unpaired t-test. **n** Left ventricular sections from *Mlc2v-cre*^*+*^ (*n* = 3) and *Mlc2v-cre*^*-*^ (*n* = 3) mice injected with AAV9-loxP-STOPcassette-loxP-miR-27b viruses were stained with H&E and imaged by light microscopy. 3 sections/heart with 3–5 fields/section were surveyed with representative fields shown. Scale bar is 200 μm. **o** Left ventricular longitudinal sections from *Mlc2v-cre*^*+*^ (*n* = 3) and *Mlc2v-cre*^*-*^ (*n* = 3) mice transduced with AAV9-loxP-STOPcassette-loxP-miR-27b were stained for DAPI (blue), laminin (green) and α-actinin (red), and imaged by confocal microscopy. 3 sections/heart with 3–5 fields/section were analyzed with representative z-stack fields shown. Arrows indicate nucleation of cardiomyocytes. Scale bar is 20 μm. **p** Heart sections stained as in (**o**) were assessed for the ratio of mononucleated to multinucleated cardiomyocytes. 3 sections/heart were analyzed with 3–5 fields/section used for quantification. In total, 3 hearts were analyzed per group. **q**, **r** Cardiomyocytes were isolated from *Mlc2v-cre*^*+*^ and *Mlc2v-cre*^*-*^ mice injected with AAV9-loxP-STOPcassette-loxP-miR-27b viruses and stained for DAPI and α-actinin (**q**), and cell size distribution of cardiomyocytes from the respective groups quantified (left panel), and differences in cardiomyocyte cell numbers with a cell size above that of the largest cardiomyocyte detected in heathy controls (**r**). Cells were imaged by confocal microscopy and a representative z-stack image from 3 mice per group is shown. Scale bar is 20 μm. *n* = 120–200 cardiomyocytes were analyzed per heart. *P < 0.05; two-tailed unpaired t-test. **s** Heart sections stained as in (**q**) were assessed for the ratio of mononucleated to multinucleated cardiomyocytes. 3 sections/heart were analyzed with 3–5 fields/section used for quantification. In total, 3 hearts were analyzed per group

In order to assess if miR-27b expression correlates with human cardiac pathology, we profiled its expression in healthy and diseased human cardiac biopsies. As noted in Fig. 3a, miR-27b expression was elevated in left ventricular biopsies of HCM and AS patients compared to healthy subjects, thus inversely correlating with ATP5A1 expression (Fig. 1a). We examined the causal association between miR-27b and Atp5a1 in vivo through delivery of AAV9 carrying pre-miR-27b (AAV9-loxP-STOP-cassette-loxP-miR-27b) to *Mlc2v-Cre* transgenic mice to achieve ectopic miR-27b expression specifically in the ventricular myocardium. Ectopic miR-27b expression was sufficient to repress *Atp5a1* mRNA and protein expression, drive pathologic cardiac overgrowth, contractile dysfunction, cardiac fibrosis and hypertrophic marker expression (Fig. 3b–j and Supplementary Table 1). Consistent with our previously identified role for ATP5A1 in pathology,^9^ an increase in the fraction of multinucleated cardiomyocytes, as readout of elevated endoreplication, was observed in left ventricles of *Mlc2v-Cre*^*+*^ mice compared to *Cre*^*–*^ littermates in sections of cardiac biopsies (Fig. 3n–p). To confirm these findings, we isolated primary adult cardiomyocytes from the respective mice and stained cells for confocal imaging and cell size and nucleation quantification. Consistent with the data above, analysis at the single-cell level revealed a shift towards more hypertrophic and multinucleated cardiomyocytes in mice expressing ectopic miR-27b, compared to controls (Fig. 3q–s).

**Fig. 4:**
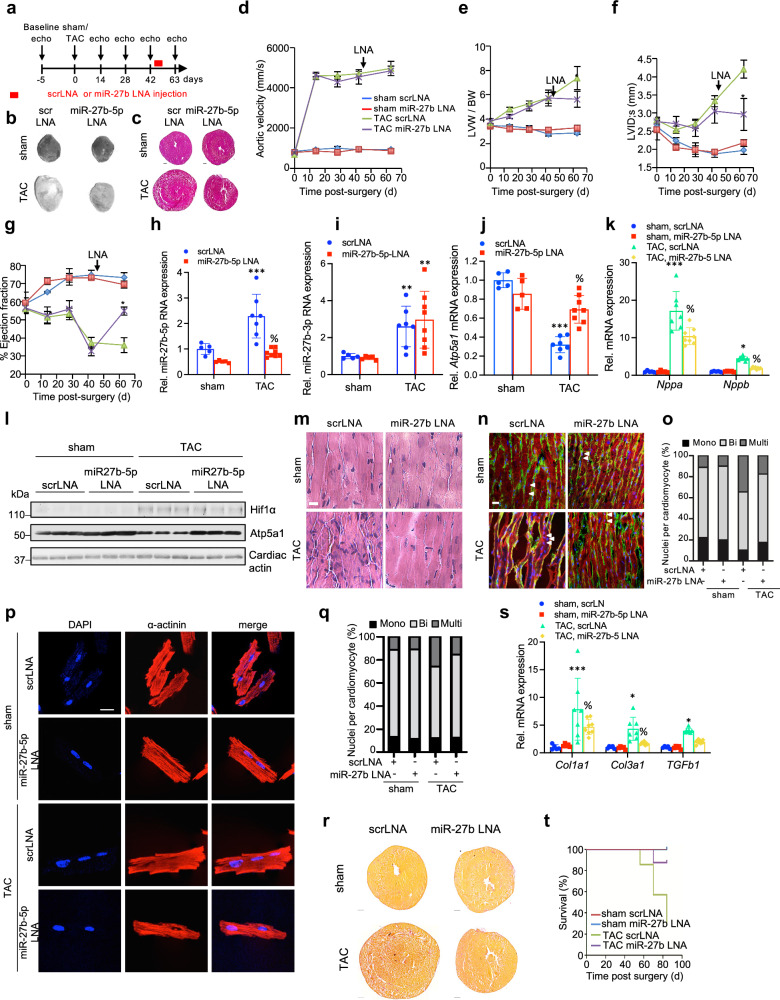
miR-27b inactivation attenuates stress-induced endoreplication and pathologic cardiac growth. **a** Schematic representation of the antagomir study in sham- and TAC-operated mice. Baseline echocardiography measurement was performed at day = −5, sham or TAC surgery at day = 0, scrLNA and miR-27b-5p LNAs delivered intraperitoneal (i.p) at day = 49–52 post-surgery and monitored by echocardiography at the indicated time points. **b**, **c** Representative images of left ventricles (**b**) and H&E-stained (**c**) histological sections. Scale bar is 1 mm. **d**–**g** Longitudinal monitoring of aortic velocity (**d**), LVW/BW (**e**), left ventricular internal diameter at end systole (LVID;s) (**f**) and ejection fraction (**g**) of C57BL/6 J mice subjected to sham or TAC surgery and treated with either scrambled (scrLNA) or miR-27b-5p LNAs. Arrows in panels indicate LNA injections (*n* = 5 for sham scrLNA, *n* = 5 for sham miR-27b-5p LNA, *n* = 7 for TAC scrLNA, *n* = 8 for TAC miR27b-5p LNA; shown is mean ± SD; * *P* < 0.05; Two-way ANOVA and Tukey’s post test). **h**–**k** Relative expression of mature miR-27b-5p (**h**), miR-27b-3p (**i**), *Atp5a1* RNA (**j**) and *Nppa* and *Nppb* RNA (**k**) in C57BL/6 J mice treated with scrLNA or miR-27b-5p LNAs and subjected to either sham or TAC surgery by qPCR. Data is normalized to sham-operated mice treated with scrLNA (set as 1.0) (*n* = 5 for sham scrLNA, *n* = 5 for sham miR-27b-5p LNA, *n* = 7 for TAC scrLNA, *n* = 8 for TAC miR-27b-5p LNA; shown is mean ± SD; *P < 0.05, **P < 0.01, ***P < 0.001 vs. sham scrLNA, % *P* < 0.01 vs. TAC scrLNA; Two-way ANOVA and Tukey’s post test). **L** Immunoblot of ventricular lysates from sham- and TAC operated C57BL/6 J mice treated with either scrLNA or miR-27b-5p LNAs using antibodies against denoted proteins. Loading is normalized to cardiac actin. **m** Left ventricular sections from C57BL/6 J mice subjected to sham or TAC surgery and treated with either scrLNA or miR-27b-5p LNAs were stained with H&E and imaged by light microscopy. 3 sections/heart with 2–4 fields/section were surveyed with representative fields shown. In total, 3 hearts were surveyed per group. Scale bar is 200 μm. **n** Left ventricular longitudinal sections from sham- or TAC operated mice treated with either scrLNA or miR-27b-5p LNAs were stained for DAPI (blue), laminin (green) and α-actinin (red), and imaged by confocal microscopy. (3 sections/heart were analyzed with 2–4 fields/section imaged with representative z-stack fields shown. In total, 3 hearts were analyzed per group). Arrows indicate nucleation of cardiomyocytes. Scale bar is 20 μm. **o** Heart sections stained as in (**n**) were assessed for the ratio of mononucleated to multinucleated cardiomyocytes. (3 sections/heart were analyzed with 2–4 fields/section used for quantification. In total, 3 hearts were analyzed per group). **p** Cardiomyocytes isolated from C57BL/6 J mice subjected to sham or TAC surgery and treated with either scrLNA or miR-27b-5p LNAs and stained for DAPI and α-actinin. Cells were imaged by confocal microscopy and a representative z-stack image from 3 mice per group is shown. Scale bar is 20 μm. **q** Adult cardiomyocytes stained as in (**p**) were assessed for the ratio of mononucleated to multinucleated cardiomyocytes. (At least 150 cells were analyzed from *n* = 3 mice per group). **r** Representative images of picrosirius red-stained histological sections of C57BL/6 J mice subjected to sham or TAC surgery and treated with either scrLNA or miR-27b-5p LNAs. Scale bar is 500 μm. **s** Relative expression of *Col1a1*, *Col3a1* and *TGFb1* mRNA in left ventricular lysates of C57BL/6 J mice treated with scrLNA or miR-27b-5p LNAs and subjected to either sham or TAC surgery by qPCR. Data is normalized to sham-operated mice treated with scrLNA (set as 1.0). *n* = 5 for sham scrLNA, *n* = 5 for sham miR-27b-5p LNA, *n* = 7 for TAC scrLNA, *n* = 8 for TAC miR27b-5p LNA; shown is mean ± SD; *P < 0.05, ***P < 0.001 vs. sham scrLNA, % *P* < 0.05 vs. TAC scrLNA; Two-way ANOVA and Tukey’s post-test. **t** Kaplan-Meier survival curves comparing mortality between mice subjected to sham or TAC surgery and treated with scrLNA or miR-27b-5p LNAs. No mortality was observed in sham-operated animals. *n* = 5 for sham scrLNA, *n* = 5 for sham miR27b-5p LNA, *n* = 7 for TAC scrLNA and *n* = 8 for TAC miR27b-5p LNA; *p* = 0.0067 Log-rank (Mantel–Cox) test

These observations and our identification of miR-27b-5p as the principal regulator of Atp5a1 expression (Fig. 2b, f–p) led us to interrogate miR-27b-5p function in mice exhibiting severe heart failure—a disease state commonly associated with ATP synthase repression and ATP depletion.^26–28^ C57BL/6 J mice were randomly assigned to two groups, with the groups subjected to either sham or TAC surgery and further subdivided into treatment groups with scrambled LNA (scrLNA) or miR-27b-5p LNA (Fig. 4a). In order to mimic late-stage human heart failure, TAC surgery was performed and hypertrophy allowed to progress till severe cardiac dysfunction was detected by echocardiography. At which time LNA treatment was initiated and echocardiography performed to monitor disease progression, as indicated in the respective figure panels (Fig. 4a–g). TAC surgery led to an increase in aortic flow velocity, a control for the degree of overload applied on the myocardium, while sham treated mice displayed normal aortic flow (Fig. 4d). Concomitant to severe cardiac hypertrophy, as demonstrated by the increased left ventricular weight: body weight ratio (LVW:BW) and ventricular dilatation, systolic cardiac dysfunction was evident by a dramatically decreased ejection fraction (EF) (Fig. 4e–g and Supplementary Table 2) in TAC mice at 42-day post-surgery. Following this observation, mice from the respective groups were subdivided for scrLNA or miR-27b-5p LNA therapy. Longitudinal monitoring of disease progression by echocardiography revealed an absence of pathology in sham operated mice. TAC operated mice treated with scrLNA displayed a further decline in cardiac function and heart failure as evidenced by pronounced hypertrophy development, ventricular dilatation and reduced cardiac ejection fraction (Fig. 4e–g and Supplementary Table 2). In contrast, TAC mice treated with miR-27b-5p LNA exhibited improved cardiac function and partial reversion of hypertrophic growth (Fig. 4e–g and Supplementary Table 2). Analysis of miR-27b-3p and miR-27b-5p expression confirmed specific inactivation of the miR-27b-5p species (Fig. 4h, i), correlating with reduced Atp5a1 mRNA and protein and elevated hypertrophic marker gene expression (Fig. 4j–l), compared to scrambled LNA treated TAC operated mice. Strikingly, the stress-induced increase in multinucleation detected by H&E and immunofluorescent staining of left ventricular sections from TAC operated mice injected with scrambled LNAs was markedly reduced in TAC operated mice receiving miR-27b-5p LNAs both in ventricular biopsy sections from these mice (Fig. 4m–o). Consistent with the data above, analysis at the single-cell level revealed a shift towards more hypertrophic and multinucleated cardiomyocytes in mice subjected to TAC, compared to controls (Fig. 4p, q and Supplementary Fig. 2a). These TAC-driven effects were largely negated upon LNA-mediated miR27b-5p inactivation (Figs. 2a and 4p, q). Likewise, miR-27b-5p LNA administration significantly reduced cardiac fibrosis and mortality in mice subjected to TAC surgery compared to controls (Fig. 4r–t). Thus, pathologic-stress induced miR-27B-5p expression and ATP5A1 repression is required for maintenance of key aspects of pathological cardiac growth in humans and mice.

To better define the underlying mechanism, we analyzed cardiomyocyte ploidy and cell size in HIF1α, miR-27b and ATP5A1 gain- and loss of function settings. As shown by imaging coupled flow cytometry and flow cytometry of propidium iodide (PI) stained DNA, ectopic HIF1α or miR-27b expression was sufficient to increase the population of multinucleated polyploid cells (Supplementary Fig. 3a–d), an effect reverted upon simultaneous miR27b-5p LNA-mediated inactivation (Supplementary Fig. 3a, b) or ectopic ATP5A1 expression (Supplementary Fig. 3c, d). In order to directly visualize endoreplicated multinucleated cells, cardiomyocytes were stained for phosphorylated histone 3 at serine 10 (p-Histone H3) and imaged by confocal microscopy. Phosphorylated histone H3 marks condensed chromosomes that are characteristic of karyokinetic cells.^29^ Consistent with the flow cytometry data, ectopic HIF1α expression led to increased phosphorylated histone H3 stained nuclei which was rescued upon parallel miR-27b-5p inhibition with LNAs (Supplementary Fig. 3e, f). Furthermore, miR-27b overexpression similarly led to increased numbers of karyokinetic cells compared to corresponding controls (Supplementary Fig. 3g, h). Remarkably, simultaneous expression of ectopic ATP5A1 and miR-27b reduced the extensive pattern of phospho-Histone H3 staining as observed upon ectopic expression of miR-27b alone (Supplementary Fig. 3g, h). To verify that the increase in de novo purine biosynthesis and multinucleation links directly to cell growth, we performed leucine-incorporation assays under identical treatment conditions. As shown in Supplementary Fig. 3i, j ectopic HIF1α, or miR-27b expression led to increased leucine-incorporation (indicative of increased protein synthesis and cell growth), which was negated upon simultaneous miR-27b-5p inactivation (Supplementary Fig. 3i), or ectopic ATP5A1 expression (Supplementary Fig. 3j). Thus, our data supports the view that reprogramming of the metabolic environment is sufficient to drive cardiomyocyte endoreplication, resulting in multinucleation and cell growth.

### HIF1α-miR-27b-5p axis activation drives purine biosynthesis

**Fig. 5:**
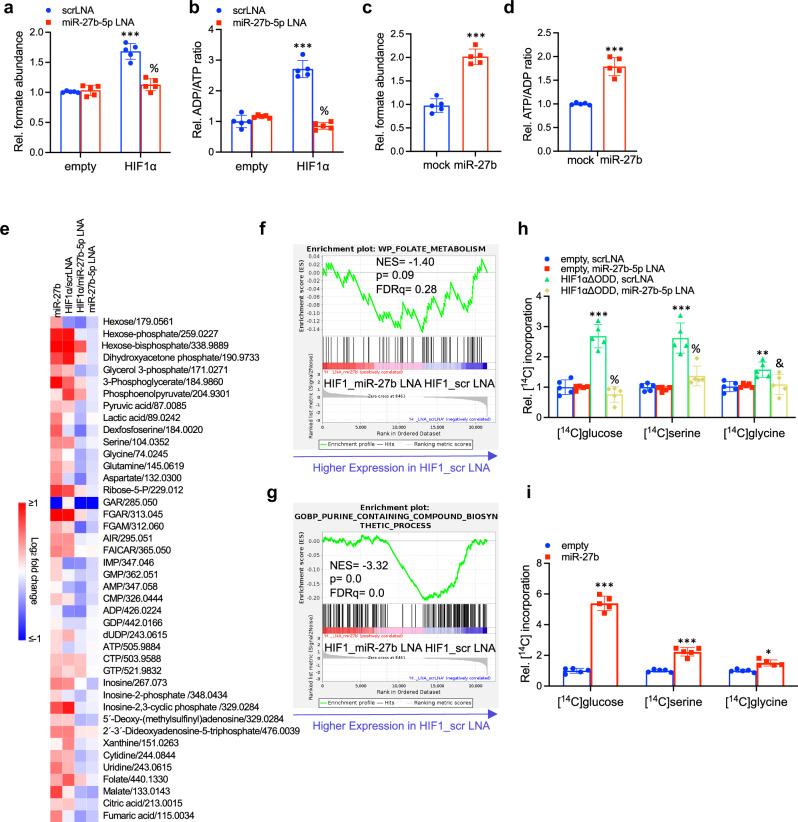
HIF1α-miR27b-5p activation promotes de novo nucleotide biosynthesis. **a**–**d** Relative amount of formate (**a**, **c**) and relative mitochondrial ADP/ATP ratio (**b**, **d**) in NRCs transduced and treated as denoted (*n* = 5 biological replicates per group; results shown are the mean ± SD; ***P < 0.001 vs. empty + scrLNA, % *P* < 0.001 vs. Hif1α+scrLNA; Two-way ANOVA followed by Tukey’s post-test (**a**, **b**); ***P < 0.001, two-tailed unpaired t-test (**c**, **d**)). **e** Heat map of relative metabolite abundance in NRCs transduced and treated as indicated. Depicted are metabolites with log_2_(fold change) > 0.5 compared to control treatment and adjusted *P* value < 0.01 in at least one treatment group compared to corresponding control (*n* = 4 biological replicates per group). **f**, **g** Enrichment plots of Gene set enrichment analyses for denoted pathways in NRCs transduced and treated as indicated (*n* = 3 biological replicates per group). **h**, **i** Relative amount of [^14^C]carbon derived from [^14^C]glucose, [^14^C]serine and [^14^C]glycine incorporated into nucleic acids in NRCs transduced and treated as denoted. *n* = 5 biological replicates per group; results shown are the mean ± SD; **P < 0.01, ***P < 0.001; % *P* < 0.001 compared to scrLNA; & *P* < 0.05 compared to scrLNA + HIF1αΔODD, Two-way ANOVA followed by Tukey’s post-test (**h**); *P < 0.05, ***P < 0.001, two-tailed unpaired t-test (**I**)

Given that ATP synthase inactivation has been shown to elevate intra-mitochondrial ADP levels^30,31^ leading to elevated purine biosynthesis and endomitosis,^9^ we explored if HIF1α and/or miR-27b-5p function could induce 1-carbon pathway activity to drive formate biosynthesis, a critical precursor and rate-limiting step for purine biosynthesis.^13,32–34^ The 1-carbon pathway utilizes ADP as a rate-limiting cofactor for the hydrolysis of 10-formyltetrahydrofolate (CHO-THF) to formate (Supplementary Fig. 4a).^12,35,36^ To test this we measured formate and mitochondrial ATP and ADP levels in genetic Hif1α and miR27b gain- and loss-of-function settings (Fig. 5a–d and Supplementary Fig. 5a–d). Increased formate generation, concomitant to reduced ATP and elevated ADP:ATP ratios, correlated directly with ectopic HIF1α or miR-27b expression (Fig. 5a–d). Next, we performed metabolomics in similar settings as above to investigate the metabolic link between activation of the HIF1α-miR-27b-ATP5A1 axis and elevated levels of multinucleation and cardiomyocyte growth (Fig. 5e). Strikingly ectopic HIF1α, or miR-27b expression resulted in increased levels of glucose and glycolytic intermediates (Dihydroxyacetonephosphate, 3-Phosphoglycerate) pointing to increased glucose uptake and glycolysis. Moreover elevated levels of serine and glycine, intermediates of the purine biosynthesis pathway (Ribose-5-phosphate, FGAR) as well as purine and pyrimidine derivatives (Inosine, Xanthine, dUTP, GTP, Uridine) were observed. In contrast, concomitant miR-27b-5p inactivation or ectopic ATP5A1 expression partially reversed these effects (Fig. 5e). Similar results were observed with the pathological hypertrophic agent T3,^37^ resulting in highly miR-27b-dependent growth, formate production and ADP/ATP increase (Supplementary Fig. 6a–c). LC-MS based analysis of the metabolome revealed increased production of purine and pyrimidine derivatives as a consequence of miR-27b expression (Supplementary Fig. 6d). Thus, mitochondrial ADP regeneration connects energetic compromise to stress-induced biosynthesis pathways. Consistent with these findings, Gene set enrichment analyses (GSEA) based on RNA-Sequencing of NRC treated with miR-27b LNA or scrLNA in the presence of HIF1α revealed an induction of folate metabolism and synthesis of purine containing compounds in scrLNA treated NRC compared to miR-27b LNA treated NRC (Fig. 5f, g). Moreover, further GSEA analyses revealed an upregulation of glycolytic and hypertrophic cardiomyopathy genes in scrLNA treated NRC compared to miR-27b LNA treated NRC (Supplementary Fig. 6e, f).

Purines are essential for nucleic acid synthesis, endoreplication and cell growth.^38,39^ Its de novo synthesis can be traced to the glucose that enters the cell and undergoes glycolysis to form 3-phosphoglycerate (3-PG), and further metabolized to generate serine and (indirectly) glycine via the serine biosynthesis pathway, which then serve as key 1-carbon donors through incorporation of its carbon into the purine ring (Supplementary Fig. 4a).^36,40,41^ As the carbon atom incorporated into the purine ring essentially traces back to glucose carbons, we followed the flux of carbon into the purine ring of nucleic acids through labeling of glucose, serine and glycine, respectively (Fig. 5h, i and Supplementary Fig. 6g). As shown, increased incorporation of carbon atoms derived from glucose, serine and glycine was observed in the nucleic acid fraction of cells ectopically expressing HIF1α or miR-27b (Fig. 5h, i), while miR27b-5p inactivation on the background of ectopic HIF1α led to pronounced suppression carbon atom incorporation (Fig. 5h), in line with the obtained metabolome profile (Fig. 5e). Similar effects were observed in settings of T3-induced cell growth and miR-27b-5p inactivation (Supplementary Fig. 6g). These data suggest that the entire upstream network impinges on the crosstalk between HIF1α and miR-27b in regulating pathology-induced de novo nucleic acid synthesis.

Finally, we determined if similar changes in metabolite distribution could be detected in left ventricular biopsies of mice subjected to either sham or TAC surgery with scrambled LNA (scrLNA) or miR-27b-5p LNA treatment (Fig. 4a–g). As noted in Supplementary Fig. 7a, the metabolite changes observed in cardiac left ventricles closely paralleled changes observed in vitro above, including the elevation of purine precursors such as N-Formylglycinamide ribonucleotide (FGAR) and 5-Formamidoimidazole-4-carboxamide ribotide (FAICAR) in scrLNA treated TAC mice compared to TAC-operated animals treated with miR-27b-5p LNA or scrLNA treated mice subjected to sham surgery (Supplementary Fig. 7a). Furthermore, the purines xanthine and inosine were significantly lower abundant in miR-27b-5p treated TAC mice compared to TAC-operated mice treated with scrLNA (Supplementary Fig. 7a). Hierarchical clustering revealed that the metabolic signature of left ventricular biopsies from TAC-treated mice injected with miR-27b-5p LNAs was altered compared to scrLNA injected TAC-mice, but comparable to that of sham-operated scrLNA and miR-27b-5p LNA treated mice (Supplementary Fig. 7b). In particular, TAC-operated left ventricular biopsies of miR-27b-5p LNA injected mice revealed lower levels of free fatty acids and other lipid species compared to TAC scrLNA controls (Supplementary Fig. 7a). Therefore, we additionally investigated the lipidome in the above-named in vivo samples (Supplementary Fig. 8a–g). Principal component analyses (PCA) indicated a unique lipidomic signature in left ventricles of TAC-operated scrLNA vs. miR-27b-5p LNA injected mice (Supplementary Fig. 8a). Lipidomes for sham- or TAC-operated scrLNA treated hearts partially overlap, but significantly separate along the first Principal Component (PC1) whereas lipidomes of sham-or TAC treated hearts with diminished miR-27b-5p expression levels significantly separate along the second principal component (PC2) (Supplementary Fig. 8b, c). A closer look into the lipid composition revealed that left ventricular biopsies of TAC-operated/miR-27b-5p LNA injected mice show indeed the highest amount of lipids containing 6 double-bonds (db), while TAC scrLNA samples significantly stand out for 2db, 4db and an accumulation for lipids with 34 and 36 carbon atoms (Supplementary Fig. 8d, e).

Analyzing the abundance of individual lipid species in TAC-operated mice *minus* mean mol% abundance of lipid species in corresponding controls revealed an enrichment of the polyunsaturated ω − 6 fatty acids arachidonic (20:4) and linoleic (18:2) acid in TAC samples (Supplementary Fig. 8f). Strikingly, lipids containing the polyunsaturated ω − 3 docosahexaenoic acid (22:6) accumulate in the left ventricle of TAC-operated mice with repressed miR-27b-5p expression (Supplementary Fig. 8g). In line with this result, free docosahexanoic acid was also increased in the untargeted metabolic analysis (Supplementary Fig. 7a). Taken together the comprehensive analysis of the cardiac lipidome revealed a correlation between the prevention of left ventricular growth in response to pressure overload and an enrichment of ω − 3 polyunsaturated lipids, especially lipids containing docosahexanoic acid, which has been shown to be cardioprotective.^42^ Increased hypertrophy on the other hand is linked with a significant upregulation of lipids containing fatty acids with 34 and 36 carbons, pointing to decreased fatty acid oxidation. In accord with the greater oxidative phenotype of hearts derived from TAC mice treated with miR-27b-5p LNA (compared to mice subjected to TAC and treated with scrLNA) (Supplementary Fig. 7a), lipid catabolism was markedly elevated in miR-27b-5p LNA-treated mice, as evidenced by enrichment of long chain fatty acids in TAC-operated hearts and treated with scrLNA compared to miR-27b-5p LNA-treated TAC hearts (Supplementary Fig. 8a–g). Furthermore, an increased expression of lipid catabolism mediators was observed in miR-27b-5p LNA-treated TAC hearts (compared to mice subjected to TAC and treated with scrLNA), corresponding to an increased palmitate oxidation capacity in miR-27b-5p LNA-treated TAC hearts (Supplementary Fig. 8h, i). In sum, these analyses revealed a unique lipid signature for miR-27b-5p LNA-treated TAC hearts characterized by a significant upregulation of lipids bearing the cardioprotective ω − 3 docosahexanoic acid and an increased fatty acid oxidation capacity, consistent with the de-repression of mitochondrial function afforded by the inactivation of miR-27b-5p.

### Methotrexate inhibits miR-27b function in pathology

**Fig. 6:**
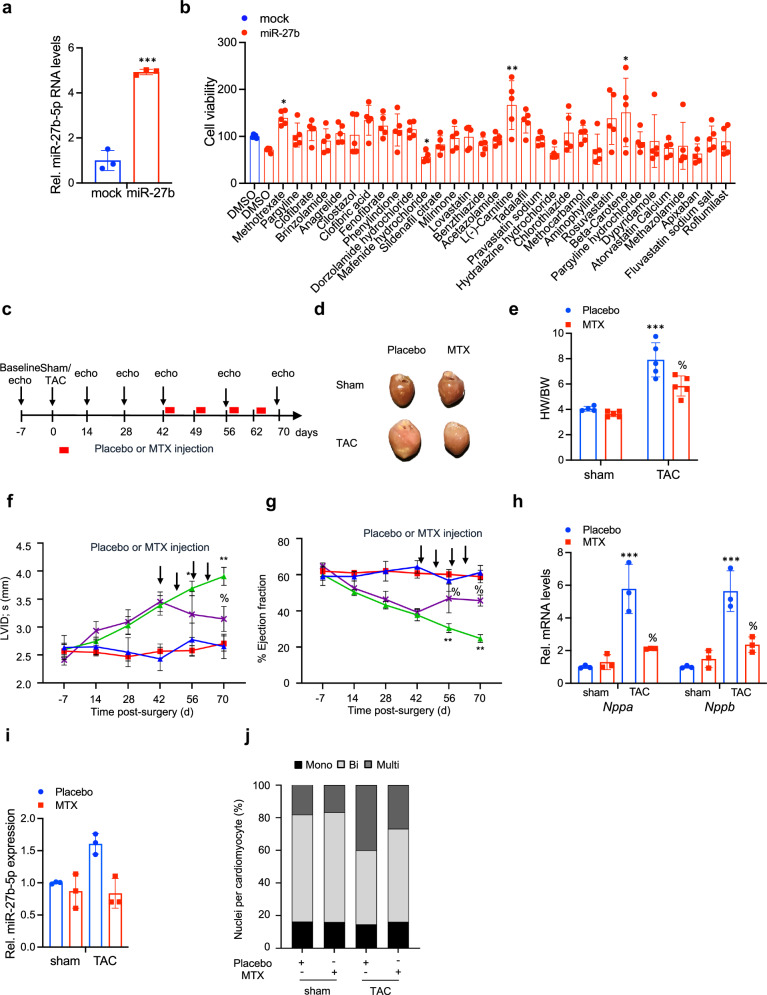
Methotrexate inhibits miR-27b function in pathology. **a** Relative expression of miR-27b in NRCs transduced with a miR-27b overexpressing construct. *n* = 3 for control, *n* = 3 for miR-27b transduced cells; results shown are the mean ± SD; **P < 0.01; two-tailed unpaired t-test. **b** Cell viability screening of FDA-approved drug subsets in NRCs ectopically expressing miR-27b. Data is normalized to the drug vehicle control Dimethyl sulfoxide (DMSO). *n* = 5–6 biological replicates per group; results shown are the mean ± SD; *P < 0.05; **P < 0.01; One-way ANOVA followed by original FDR method by Benjamini and Hochberg. **c** Schematic representation of the experimental timeline of sham and TAC mice treated with the Placebo or MTX, respectively. **d** Representative images of hearts of sham and TAC mice treated mice, administered the Placebo or MTX, respectively. **e**–**g** HW/BW (**e**), left ventricular internal diameter at systole (LVID;s) (**f**) and ejection fraction (**g**) of sham and TAC mice administered the Placebo or MTX, respectively. Arrows in panels indicate Placebo or MTX injection (*n* = 4 for sham Placebo, *n* = 4 for sham MTX, *n* = 5 for TAC Placebo, *n* = 6 for TAC Methotrexate). **h** Relative expression of *Nppa* and *Nppb* in C57BL/6 J mice subjected to sham or TAC surgery and injected with either Placebo or MTX. **i** Relative expression level of miR-27b in C57BL/6 J mice subjected to sham or TAC surgery and injected with either Placebo or MTX. **j** quantification of the ratio of mononucleated, binucleated and multinucleated cardiomyocytes. 3 sections/heart were analyzed with 3–5 fields/section used for quantification. In total, 3 hearts were analyzed per group. Data are represented as Mean ± SD; ***P < 0.001 vs. sham Placebo. % *P* < 0.05 vs. TAC Placebo, Two-Way ANOVA followed by Tukey’s post-test. MTX methotrexate, HW heart weight, BW body weight

Given the detrimental impact of miR-27b and its potential widespread role across different cardiac indications - as a means for direct clinical translation and repositioning, we asked if it would be possible to identify clinically approved compounds to attenuate miR-27b function and potentially phenocopy the impact of LNA-based miR-27b inhibition. We sourced our compounds from the FDA-approved drug library (comprising 2485 unique compounds), given the impact of HIF1α-miR-27b-5p pathway activation on cardiac metabolism, we refined the FDA-approved drug list to a total of 142 compounds affecting cellular metabolism, of which 32 compounds fell into the Cardiovascular Disease (CVD) category among others. Constitutive mir27b expression is toxic and drives cardiomyocyte cell death (Fig. 6a, b).^43^ Thus, in utilizing cell viability as readout we screened the 32-drug subset for their capacity to inhibit miR-27b-mediated cardiomyocyte death (Fig. 6a, b). Of these, four compounds including methotrexate, clofibric acid, fenofibrate and carnitine proved most effective in inhibiting miR-27b-induced cell death. With the exception of methotrexate, all other top candidates have previously been tightly linked to improved cardiac (and cardiac mitochondrial) function via activation of the peroxisome proliferator-activated receptor alpha (PPARα) pathway (clofibric acid, fenofibrate), or to activation of mitochondrial fatty acid transport (carnitine). While interesting, we focused on methotrexate (MTX) whose role in cardiac and mitochondrial function is less established. MTX is clinically used (at different dosages) for treatment of cancers such as leukemia and certain sub-types of breast and skin cancers, and more recently for autoimmune disease, including psoriasis and rheumatoid arthritis.^44^ Mechanistically, MTX functions as a folate analog with a 1000-fold greater affinity for its target, dihydrofolate reductase (DHFR), than folate, thus serving as DHFR competitive inhibitor.^45^ DHFR is a rate-limiting enzyme of purine biosynthesis, catalyzing the conversion of dihydrofolate to the tetrahydrofolate, a critical precursor for purine biosynthesis.^45^

Given our observations that miR-27b can induce aberrant purine biosynthesis leading to endomitosis and cardiac hypertrophy, we reasoned that an anti-folate such as MTX might serve to prevent induction of cardiac endomitosis and pathologic growth, and possibly phenocopy the effects of LNA-mediated miR-27b inhibition. To that end, C57BL/6 J mice were randomly assigned to two groups, with the groups subjected to either sham or TAC surgery and further subdivided into treatment groups with Placebo (vehicle control) or MTX. Due to its pharmacokinetics and biodistribution,^46^ 42 days post-TAC surgery, MTX was delivered subcutaneously weekly as depicted in Fig. 6c. TAC surgery led to an increase in aortic flow velocity, while sham treated mice displayed normal flow levels (Supplementary Fig. 9a). TAC operated mice treated with placebo displayed declined cardiac function as evidenced by pronounced cardiac hypertrophy, ventricular dilatation and reduced cardiac ejection fraction, and elevated hypertrophic marker expression (Fig. 6d–h). In contrast, TAC operated mice treated subcutaneously on weekly basis with MTX exhibited significant reduction in ventricular wall hypertrophy and lumen dilation, concomitant to improved cardiac function as evidenced by blunted *Nppa* and *Nppb* expression and maintenance of cardiac function, at near sham control levels, despite the significant stress imposed on the heart as a result of aortic constriction (Fig. 6d–h and Supplementary Fig. 9a).

As previously observed, TAC surgery led to elevated miR-27b-5p levels in left ventricular biopsies, compared to sham controls (Figs. 1i, j, n and 4h). Surprisingly, when we assessed mice subjected to TAC surgery and administered MTX, we detected a specific suppression of miR-27b-5p RNA (Fig. 6i). We suspected this observation being link to MTX mediated suppression of purine biosynthesis and endomitosis, and as consequence pathologic growth. Thus, in the absence pathologic growth, equilibrium of the vascular network is maintained and intra-tissue hypoxia development curtailed—leading to absence of HIF1α activation and downstream miR-27b transcriptional induction. Furthermore, the stress-induced increase in multinucleation detected by immunofluorescent staining of left ventricular sections from TAC operated mice injected with Placebo was markedly reduced in TAC operated mice receiving MTX (Fig. 6j and Supplementary Fig. 9b). Importantly however, is the fact that MTX administration blunted the induction endomitosis, pathological growth and of cardiac dysfunction, in accord with previous findings related to ectopic miR-27b expression and to LNA-mediated miR27b inhibition. Thus, MTX serves to inhibit pathologic endoreplication and cardiac hypertrophy, likely through its dual impact on the purine biosynthesis pathway.

Pathological hypertrophy has long been linked to impaired mitochondrial energetics, yet the upstream regulatory cues that connect hypoxia to mitochondrial dysfunction remained undefined. In this study, we identify HIF1α as the transcriptional activator of miR-27b-5p, establishing a direct link between microregional hypoxia and mitochondrial ATP synthase repression. HIF1α binding to a conserved promoter element selectively induced miR-27b-5p, while sparing miR-27b-3p and its cluster partners, thereby revealing strand- and stimulus-specific regulation. This induction was sufficient to suppress ATP5A1, disrupt mitochondrial bioenergetics, and drive maladaptive cardiomyocyte growth. Therapeutically, systemic LNA inhibition of miR-27b-5p restored ATP5A1, reversed multinucleation and fibrosis, and improved ventricular function in pressure-overloaded hearts. Strikingly, the antifolate methotrexate, by constraining purine biosynthesis, phenocopied anti-miR-27b-5p therapy, relieving hypoxia and attenuating remodeling. These findings delineate a previously unrecognized HIF1α-miR-27b-5p-ATP5A1 axis and identify complementary molecular and pharmacological strategies to intercept it.

## Discussion

In its essence, our study identifies a conserved mechanistic pathway driving endoreplication and growth control in hypertrophic heart disease. Mechanistically, these effects are provoked by pathologic stimuli through HIF1α activation of miR-27b-mediated repression of the alpha subunit of ATP synthase (ATP5A1) to facilitate purine biosynthesis, karyokinesis and cardiac growth.

MiR-27b was identified as a HIF1α-regulated miRNA from genome-wide miRNA expression studies in mouse models of cardiac hypertrophy and dysfunction (Fig. 1i–v). Furthermore, gain- and loss-of-function studies in mice demonstrated that miR-27b is necessary and sufficient for the development and maintenance of heart disease. Ectopic miR-27b expression in the ventricle of the heart promoted pathologic cardiac growth as evidenced by increased left ventricular weight in mice ectopically expressing miR-27b compared to control mice (Fig. 3c–e, q, r). Moreover, miR-27b expression promoted contractile dysfunction as observed by decreased ejection fraction and increased left ventricular internal diameter (Fig. 3f). These findings are in line with a previous report demonstrating the development of cardiac hypertrophy in transgenic mice overexpressing miR-27b.^47^ In contrast, inhibition of miR-27b with LNAs in TAC-operated animals prevented the contractile dysfunction observed in scrambled-LNA treated mice in similar settings (Fig. 4b, c, e, p and Supplementary Fig. 2a). Moreover, administration of miR-27b targeting LNAs after mice had progressed to heart failure reversed pathologic progression, and improved contractility and pathologic growth regression (Fig. 4d–g). These data indicate that miR-27b is required for the development and maintenance of the pathology. Consistent with these findings, Wang and colleagues previously reported a beneficial effect of miR-27b inactivation in mice subjected to TAC.^47^

Despite being encoded within the miR-23b-27b-24 cluster, HIF1α and other pathologic stressors such as TAC surgery and T3 treatment promoted miR-27b expression independently from both its miRNA cluster and host gene *Aopep*. While the miR-23b/27b/24-1 cluster can be co-transcribed as a single unit in certain tissues and conditions, our findings demonstrate that in the hypoxic heart miR-27b is transcriptionally upregulated via a dedicated HIF1α-responsive promoter, independent of miR-23b and miR-24, resulting in its selective induction without concomitant cluster member activation.^48,49^ Hence, these stressors induce mature miR-27b expression while mature miR-23b, miR-24 and *Aopep* expression levels remained unchanged (Fig. 1l–q, and Supplementary Fig. 1d–f). miR-24 expression does not represent the miR-24 expression from the miR-23b-27b-24 cluster alone but rather reflects the miR-24 expression from both the miR-23b-27b-24 on mouse chromosome 13 and miR-23a-27a-24 cluster encoded on mouse chromosome 8.^22,50^ It is impossible to discriminate between miR-24-1 and miR-24-2, derived from chromosome 13 and 8, respectively, by TaqMan qRT-PCR as they bear the same sequence and are 100% homologous.^51^ Consistent with the observed independent regulation of the miRNAs in the cluster, Sun and colleagues reported that pri-miR-23b, pri-miR-27b and pri-miR-24-1 can be transcribed independently and show unique expression profiles even though they are located in close proximity.^25^ Recently, a novel miRNA, miR-3074-1, was identified from genome-wide transcriptomics data and annotated to the miR-23b-27b-24 loci.^52^ miR-3074-1 fully overlaps with miR-24-1 but is encoded by the opposite antisense strand. However, to date it remains unclear if miR-3074-1 confers biological effects or if it represents a minor non-functional species of miR-24-1.^52^

Biopsies of cardiomyopathy patients demonstrate increased expression of HIF1α and MIR27B with concordant downregulation of ATP5A1. The critical role of F_1_F_0_ ATP synthase function in mediating cell growth is supported by clinical reports of various complex V mutations, including ATP5A1, that are associated with hypertrophic cardiomyopathy and heart failure.^53,54^ Since aortic stenosis and hypertrophic cardiomyopathies are frequent morbidities in humans^55,56^ and these patients tend to have higher nucleic acid synthesis rates,^57^ the impact of the HIF1α-miR-27b-ATP5A1 axis in these patients might be of critical importance for disease progression. In line with our previous findings,^6,7^ the identification of cardiometabolic endoreplication represents a novel output of HIF1α function in linking energetic deregulation to increased DNA and RNA synthesis, thus facilitating pathological growth progression to heart failure.

The criticality of de novo nucleotide synthesis in pathologic cardiac growth is further highlighted by the fact that miR-27b-5p inactivation prevents TAC-induced cardiac hypertrophy and dysfunction. Given that the 1-carbon cycle operates predominantly in a clock-wise feed-forward manner and that the precursors of de novo purine biosynthesis lie upstream of the methylation reactions precursors,^13^ suggests a possible prioritization of purine biosynthesis over the methyl cycle with respect to the 1-carbon unit incorporation. Taken together, our findings implicate de novo purine synthesis as an important downstream component of increased mitochondrial formate synthesis, at least in heart disease. This might be of critical importance in hypertrophic or highly proliferating cells as acceleration of de novo nucleotide synthesis correlates and is necessary to support these growth scenarios.^58^ In accord, tracer experiments with the 1-carbon donors serine and glycine as well as glucose (an upstream serine precursor (Supplementary Fig. 4a), and gene set expression analyses demonstrate that the de novo nucleic acid synthesis rate is elevated upon activation of HIF1α-miR-27b-ATP5A1 pathway. Whist our study has focused on the function of miR-27b-5p and did not observe miR-27b-3p-mediated effects on ATP5A1 or bioenergetics in cardiomyocytes, miR-27b-3p may have other targets and effects in the heart as has previously been shown.^43,47,59^ Critically, systemically delivered LNAs are known to achieve meaningful uptake and target knockdown in the heart, consistent with our observation of ATP5A1 de-repression and improved cardiac function. While some uptake by non-myocytes such as fibroblasts is possible and may contribute to the anti-fibrotic response, the central effects are best explained by direct inhibition of miR-27b-5p in cardiomyocytes. Thus, the remodeling benefits most likely reflect on-target actions within cardiac cell populations rather than off-target systemic effects

As a direct consequence of elevated nucleic acid synthesis, an increase in the multinucleated cell populations was observed upon induction of the identified network. In line with these findings, cell cycle re-entry and progression is required for the development of cardiac pathology.^60–62^ Despite progression through the cell cycle and the upregulation of many proteins involved in the actomyosin ring formation for cytokinesis in the diseased state, adult cardiomyocytes are post-mitotic with minimal proliferative capacity.^63,64^ Instead, hypertrophic cardiomyocytes become multinucleated as they undergo karyokinesis but fail to complete cytokinesis.^64^ Our link between hypertrophy and endoreplication is further supported in animal models of cardiac hypertrophy and human patients with hypertrophic hearts.^26–28^ Cardiomyocyte polyploidization and the resulting increase in gene copy number has been proposed to support and drive the acceleration of metabolism and biosynthetic rate to provide macromolecules necessary to support hypertrophic growth.^65–69^ While human and mouse cardiomyocytes differ in their structural outcomes of DNA synthesis, with humans favoring polyploidization and mice multinucleation, the upstream signaling pathways and metabolic mechanisms driving pathological DNA replication appear to be highly conserved, supporting the translational relevance of our findings across species.^70^

Treatment with the folic acid antagonist MTX provided the proof of principle that functional and morphologic remodeling in pressure overload heart disease can be attenuated by inhibiting de novo purine biosynthesis and thus pathologic endoreplication. While we do not propose that methotrexate directly represses miR-27b-5p transcription, our data support the view that MTX constrains folate-dependent purine biosynthesis, thereby attenuating pathological cardiomyocyte growth and relieving myocardial hypoxia. The consequent reduction of HIF-1α activity secondarily lowers miR-27b-5p expression, thus phenocopying the effects of anti-miR-27b therapy. MTX is an inexpensive, widely used and safe drug in rheumatology. Retrospective meta-analyses have revealed a 28% risk reduction for adverse cardiovascular (CV) events and a 19% reduced risk for myocardial infarction (MI) in low-dose MTX treated patients with rheumatoid arthritis and psoriatic arthritis compared to patients who received other therapies or placebo.^71^ Although these findings have been challenged by recent findings from the Cardiovascular Inflammation Reduction Trial (CIRT),^72^ it should be emphasized that no clinical trial so far has focused specifically on a significant sub-fraction of patients with pressure-overload induced systolic heart failure with reduced ejection fraction (HFrEF). Especially in such a significant patient cohort, inhibiting purine biosynthesis by MTX could be an extremely efficient treatment opportunity to reduce cardiovascular death by reducing pathologic growth and left ventricular remodeling via preventing myocardial endoreplication. While the HIF1α-miR-27b-5p axis is certainly amenable to inhibition by more experimental gene therapy-type approaches, we propose that the identification of Methotrexate provides a more immediate route to clinics for alleviation of human heart disease. Critically, given the cost-effective nature of the compound, its storage and transport stability at ambient conditions, and its straightforward oral route of administration, we propose a potentially beneficial impact of its global use, particularly in developing nations with limited healthcare budgets and treatment infrastructure.

Taken together, our findings raise the intriguing possibility of therapeutic cardiac morphology and function renormalization through modulation of energy metabolism to restore ATP levels. This would serve to not only improve cardiac dysfunction by providing energy substrates to the contractile apparatus but would directly prevent the associated changes in cardiac morphology and growth. Given that our results suggest a role for stress-induced metabolic activation of endoreplication enforcing anabolic growth as a crucial component of the hypoxic response, these findings are likely to be of significant relevance in other pathologies such as cancer or diabetes, which are also characterized by maladaptive hypoxic responses. Thus, the activation of the HIF1α-miR-27b-ATP5A1 axis represents a critical pathway underlying the molecular and metabolic basis of endomitosis and growth control in disease.

Although mitochondrial dysfunction is a well-established hallmark of pathological hypertrophy, the proximal signals that connect hypoxia to mitochondrial failure and growth reprogramming have remained obscure. Here, we uncover a direct regulatory axis in which HIF1α activates miR-27b-5p through a dedicated promoter, linking microregional oxygen stress to repression of ATP5A1 and destabilization of mitochondrial energetics. This finding provides strand-specific resolution to the role of miR-27b, demonstrating that the 5p arm, not the typically more abundant 3p species, drives maladaptive remodeling in cardiomyocytes. Beyond mechanism, our study highlights two complementary therapeutic avenues: first, LNA-mediated inhibition of miR-27b-5p reversed established disease, underscoring the tractability of direct microRNA targeting in the failing heart; and second, the observation that methotrexate, a clinically approved antifolate, phenocopies anti-miR-27b-5p therapy reveals an unexpected opportunity for drug repurposing through metabolic modulation. Collectively, these insights define a hypoxia-microRNA-mitochondrial circuit that is both mechanistically novel and therapeutically actionable, offering new strategies to intercept cardiac remodeling.

## Materials and methods

### Animal breeding and maintenance

*Hif1α fl/fl* mice were obtained from Randall S. Johnson (University of California, San Diego, USA) and *Vhl fl/fl* mice were kindly provided by Rudolf Jaenisch (Massachusetts Institute of Technology, USA). The myosin light-chain (*Mlc*)*2v-Cre*^73^ line was from Ju Chen (University of California, San Diego, USA). The respective ventricular-specific mouse lines described in this manuscript were generated by crossing loxP-flanked *Hif1α (Hif1α fl/fl)*^74^ *or Vhl (Vhl fl/fl)*^75^ mice to myosin light-chain *Mlc2v*-*Cre* transgenic mice. The data presented in this manuscript represents studies with male mice aged 3–20 weeks of the C57BL/6 J background. By 3–20 weeks we were referring to the whole duration of the animal experiments. The AAV9 viruses were administered to 3-week-old mice. As the AAV9 vectors have a lag phase of approximately 6–10 weeks until it reaches maximal expression,^7^ surgeries were performed at 9–11 weeks and hearts harvested at least 11 weeks after AAV9 administration as shown in the experimental outlines in Fig. 3b. In TAC experiments, mice were 10–12 weeks old at the beginning of the experiment. Only mice of a similar age (+/−1 week) were used in the corresponding experiments. In experiments with the *Mlc2v-cre* mice, littermates and mice of a similar age (+/−1 week) were used. After baseline echocardiography mice were randomly assigned to groups, AAV injections, LNA delivery, methotrexate versus placebo treatment and echocardiography was performed blinded. In experiments including TAC surgery experiments were kept blinded over the baseline echocardiography until operation. All mice were maintained at the MRC Clinical Sciences Centre (Imperial College London), Institute of Molecular Health Sciences (ETH Zurich) and/or the Cardiovascular Assessment Facility (CAF), Department of Medicine, Department of Medicine, University of Lausanne in a specific pathogen-free facility. Maintenance and animal experimentation were in accordance with the Swiss Federal Veterinary Office (BVET) guidelines.

### Human and mouse ventricular biopsies

Human heart biopsies and clinical data were generously provided by Samuel Sossalla (*Georg-August-University Goettingen and DZHK*, *Goettingen*, Germany) and Sebastian Stehr (University Hospital Leipzig, Germany). Human HCM and aortic stenosis biopsies were conducted in compliance with the local ethics committee, and written informed consent was received from all subjects prior to inclusion. Myocardial samples were obtained from patients with severe aortic stenosis undergoing aortic valve replacement and a Morrow resection from the hypertrophied left ventricular septum. Only patients without significant aortic valvular regurgitation and with preserved contractile function were included. Human left ventricular biopsies of HCM patients were obtained from left ventricular papillary muscle of explanted hearts. The myocardial samples were acquired directly in the operating room during the surgery and immediately washed in precooled cardioplegic solution (110 mM NaCl, 16 mM KCl, 16 mM MgCl_2_, 16 mM NaHCO_3_, 1.2 mM CaCl_2_, 11 mM glucose) followed by rapid snap-freezing in liquid nitrogen. Healthy heart samples were obtained from left ventricles of donor hearts. Sample weight was approximately 20–150 mg. Clinical data pertaining to these subjects are shown in a previous publication.^7^

### Ethics statement and consent to participate overview

All animal experiments were conducted in accordance with institutional and national guidelines for the care and use of laboratory animals and were approved by the Home Office, United Kingdom and the Swiss Federal Veterinary Office (BVET). All procedures complied with applicable English, Swiss and European regulations governing animal experimentation.

Human cardiac tissue and clinical samples were obtained in accordance with the Declaration of Helsinki and were approved by Georg-August-Universität Goettingen, Germany. Written informed consent was obtained from all participants or their legal representatives prior to sample collection. All human data were analyzed in anonymized or pseudonymized form.

## Supplementary information

Supplementary Material

Raw data

## Data Availability

The data supporting the findings of this study are available within the article and its Supplementary Information files. Source data underlying the main and supplementary figures are available from the corresponding author upon reasonable request. Any additional datasets generated during the current study are available from the corresponding author on reasonable request and subject to applicable ethical and data protection regulations.

## References

[1] Hill, J. A. & Olson, E. N. Cardiac plasticity. *N. Engl. J. Med.* **358**, 1370–1380 (2008). 18367740 10.1056/NEJMra072139

[2] Folkman, J. How is blood vessel growth regulated in normal and neoplastic tissue? G.H.A. Clowes memorial Award lecture. *Cancer Res.* **46**, 467–473 (1986). 2416426

[3] MacLellan, W. R. & Schneider, M. D. Genetic dissection of cardiac growth control pathways. *Annu. Rev. Physiol.* **62**, 289–319 (2000). 10845093 10.1146/annurev.physiol.62.1.289

[4] Kakinuma, Y. et al. Novel molecular mechanism of increased myocardial endothelin-1 expression in the failing heart involving the transcriptional factor hypoxia-inducible factor-1alpha induced for impaired myocardial energy metabolism. *Circulation* **103**, 2387–2394 (2001). 11352889 10.1161/01.cir.103.19.2387

[5] Lee, S. H. et al. Early expression of angiogenesis factors in acute myocardial ischemia and infarction. *N. Engl. J. Med.* **342**, 626–633 (2000). 10699162 10.1056/NEJM200003023420904

[6] Krishnan, J. et al. Activation of a HIF1alpha-PPARgamma axis underlies the integration of glycolytic and lipid anabolic pathways in pathologic cardiac hypertrophy. *Cell Metab.* **9**, 512–524 (2009). 19490906 10.1016/j.cmet.2009.05.005

[7] Mirtschink, P. et al. HIF-driven SF3B1 induces KHK-C to enforce fructolysis and heart disease. *Nature* **522**, 444–449 (2015). 26083752 10.1038/nature14508 PMC4783869

[8] Bekeredjian, R. et al. Conditional HIF-1alpha expression produces a reversible cardiomyopathy. *PLoS ONE* **5**, e11693 (2010). 20657781 10.1371/journal.pone.0011693 PMC2908132

[9] Bischof, C. et al. Mitochondrial–cell cycle cross-talk drives endoreplication in heart disease. *Sci. Transl. Med.* **13**, eabi7964 (2021). 10.1126/scitranslmed.abi7964 34878823

[10] von Ballmoos, C., Wiedenmann, A. & Dimroth, P. Essentials for ATP synthesis by F1F0 ATP synthases. *Annu Rev. Biochem* **78**, 649–672 (2009). 19489730 10.1146/annurev.biochem.78.081307.104803

[11] Seagroves, T. N. et al. Transcription factor HIF-1 is a necessary mediator of the Pasteur effect in mammalian cells. *Mol. Cell. Biol.* **21**, 3436–3444 (2001). 11313469 10.1128/MCB.21.10.3436-3444.2001 PMC100265

[12] Momb, J. et al. Deletion of Mthfd1l causes embryonic lethality and neural tube and craniofacial defects in mice. *Proc. Natl. Acad. Sci. USA* **110**, 549–554 (2013). 23267094 10.1073/pnas.1211199110 PMC3545794

[13] Pike, S. T., Rajendra, R., Artzt, K. & Appling, D. R. Mitochondrial C1-tetrahydrofolate synthase (MTHFD1L) supports the flow of mitochondrial one-carbon units into the methyl cycle in embryos. *J. Biol. Chem.* **285**, 4612–4620 (2010). 19948730 10.1074/jbc.M109.079855 PMC2836066

[14] Barrick, C. J., Rojas, M., Schoonhoven, R., Smyth, S. S. & Threadgill, D. W. Cardiac response to pressure overload in 129S1/SvImJ and C57BL/6J mice: temporal- and background-dependent development of concentric left ventricular hypertrophy. *Am. J. Physiol. Heart Circ. Physiol.* **292**, H2119–H2130 (2007). 17172276 10.1152/ajpheart.00816.2006

[15] el Azzouzi, H. et al. The hypoxia-inducible microRNA cluster miR-199a approximately 214 targets myocardial PPARdelta and impairs mitochondrial fatty acid oxidation. *Cell Metab.* **18**, 341–354 (2013). 24011070 10.1016/j.cmet.2013.08.009

[16] deAlmeida, A. C., van Oort, R. J. & Wehrens, X. H. T. Transverse aortic constriction in mice. *J. Vis. Exp.* **38**, 1729 (2010). 10.3791/1729 PMC3164086 20410870

[17] Wenger, R. H., Stiehl, D. P. & Camenisch, G. Integration of oxygen signaling at the consensus HRE. *Sci. STKE* **2005**, re12 (2005). 16234508 10.1126/stke.3062005re12

[18] Blick, C. et al. Identification of a hypoxia-regulated miRNA signature in bladder cancer and a role for miR-145 in hypoxia-dependent apoptosis. *Br. J. Cancer* **113**, 634–644 (2015). 26196183 10.1038/bjc.2015.203 PMC4647685

[19] Huang, X. et al. Hypoxia-inducible miR-210 regulates normoxic gene expression involved in tumor initiation. *Mol. Cell* **35**, 856–867 (2009). 19782034 10.1016/j.molcel.2009.09.006 PMC2782615

[20] Lee, S. W. et al. MicroRNA-26a induced by hypoxia targets HDAC6 in myogenic differentiation of embryonic stem cells. *Nucleic Acids Res.* **43**, 2057–2073 (2015). 25662604 10.1093/nar/gkv088 PMC4344521

[21] Agrawal, R. et al. Hypoxic signature of microRNAs in glioblastoma: insights from small RNA deep sequencing. *BMC Genomics* **15**, 686 (2014). 25129238 10.1186/1471-2164-15-686 PMC4148931

[22] Zhou, Q. et al. Regulation of angiogenesis and choroidal neovascularization by members of microRNA-23~27~24 clusters. *Proc. Natl. Acad. Sci. USA* **108**, 8287–8292 (2011). 21536891 10.1073/pnas.1105254108 PMC3100947

[23] Huang, L. E., Gu, J., Schau, M. & Bunn, H. F. Regulation of hypoxia-inducible factor 1alpha is mediated by an O2-dependent degradation domain via the ubiquitin-proteasome pathway. *Proc. Natl. Acad. Sci. USA* **95**, 7987–7992 (1998). 9653127 10.1073/pnas.95.14.7987 PMC20916

[24] Moretto, F. C. et al. Triiodothyronine (T3) induces HIF1A and TGFA expression in MCF7 cells by activating PI3K. *Life Sci.* **154**, 52–57 (2016). 27094789 10.1016/j.lfs.2016.04.024

[25] Sun, F. et al. Characterization of function and regulation of miR-24-1 and miR-31. *Biochem. Biophys. Res. Commun.* **380**, 660–665 (2009). 19285018 10.1016/j.bbrc.2009.01.161

[26] Ashrafian, H., Redwood, C., Blair, E. & Watkins, H. Hypertrophic cardiomyopathy:a paradigm for myocardial energy depletion. *Trends Genet* **19**, 263–268 (2003). 12711218 10.1016/S0168-9525(03)00081-7

[27] Mahmod, M. et al. Myocardial perfusion and oxygenation are impaired during stress in severe aortic stenosis and correlate with impaired energetics and subclinical left ventricular dysfunction. *J. Cardiovasc. Magn. Reson* **16**, 29 (2014). 24779370 10.1186/1532-429X-16-29 PMC4009072

[28] Taha, M. & Lopaschuk, G. D. Alterations in energy metabolism in cardiomyopathies. *Ann. Med.* **39**, 594–607 (2007). 17934906 10.1080/07853890701618305

[29] Hans, F. & Dimitrov, S. Histone H3 phosphorylation and cell division. *Oncogene* **20**, 3021–3027 (2001). 11420717 10.1038/sj.onc.1204326

[30] Akerboom, T. P., Bookelman, H., Zuurendonk, P. F., van der Meer, R. & Tager, J. M. Intramitochondrial and extramitochondrial concentrations of adenine nucleotides and inorganic phosphate in isolated hepatocytes from fasted rats. *Eur. J. Biochem. / FEBS* **84**, 413–420 (1978). 10.1111/j.1432-1033.1978.tb12182.x 639797

[31] Schild, L., Blair, P. V., Davis, W. I. & Baugh, S. Effect of adenine nucleotide pool size in mitochondria on intramitochondrial ATP levels. *Biochim. Biophys. Acta* **1413**, 14–20 (1999). 10524260 10.1016/s0005-2728(99)00074-2

[32] Anguera, M. C. et al. Regulation of folate-mediated one-carbon metabolism by 10-formyltetrahydrofolate dehydrogenase. *J. Biol. Chem.* **281**, 18335–18342 (2006). 16627483 10.1074/jbc.M510623200

[33] Gregory, J. F. 3rd et al. Primed, constant infusion with [2H3]serine allows in vivo kinetic measurement of serine turnover, homocysteine remethylation, and transsulfuration processes in human one-carbon metabolism. *Am. J. Clin. Nutr.* **72**, 1535–1541 (2000). 11101483 10.1093/ajcn/72.6.1535

[34] Herbig, K. et al. Cytoplasmic serine hydroxymethyltransferase mediates competition between folate-dependent deoxyribonucleotide and S-adenosylmethionine biosyntheses. *J. Biol. Chem.* **277**, 38381–38389 (2002). 12161434 10.1074/jbc.M205000200

[35] Brosnan, M. E. & Brosnan, J. T. Formate: the neglected member of one-carbon metabolism. *Annu Rev. Nutr.* **36**, 369–388 (2016). 27431368 10.1146/annurev-nutr-071715-050738

[36] Tibbetts, A. S. & Appling, D. R. Compartmentalization of Mammalian folate-mediated one-carbon metabolism. *Annu Rev. Nutr.* **30**, 57–81 (2010). 20645850 10.1146/annurev.nutr.012809.104810

[37] Dillmann, W. Cardiac hypertrophy and thyroid hormone signaling. *Heart Fail. Rev.* **15**, 125–132 (2009). 19125327 10.1007/s10741-008-9125-7 PMC2820695

[38] Lloyd, A. C. The regulation of cell size. *Cell* **154**, 1194–1205 (2013). 24034244 10.1016/j.cell.2013.08.053

[39] Zimmer, H. G., Trendelenburg, C., Kammermeier, H. & Gerlach, E. De novo synthesis of myocardial adenine nucleotides in the rat. Acceleration during recovery from oxygen deficiency. *Circ. Res.* **32**, 635–642 (1973). 4713206 10.1161/01.res.32.5.635

[40] Jain, M. et al. Metabolite profiling identifies a key role for glycine in rapid cancer cell proliferation. *Science* **336**, 1040–1044 (2012). 22628656 10.1126/science.1218595 PMC3526189

[41] Tong, X., Zhao, F. & Thompson, C. B. The molecular determinants of de novo nucleotide biosynthesis in cancer cells. *Curr. Opin. Genet Dev.* **19**, 32–37 (2009). 19201187 10.1016/j.gde.2009.01.002 PMC2707261

[42] Endo, J. & Arita, M. Cardioprotective mechanism of omega-3 polyunsaturated fatty acids. *J. Cardiol.* **67**, 22–27 (2016). 26359712 10.1016/j.jjcc.2015.08.002

[43] Hou, N. et al. Cardiomycyte overexpression of miR-27b resulted in cardiac fibrosis and mitochondria injury in mice. *Hereditas* **34**, 326–334 (2012). 22425951 10.3724/sp.j.1005.2012.00326

[44] Goodsell, D. S. The molecular perspective: methotrexate. *Oncologist* **4**, 340–341 (1999). 10476546

[45] Rajagopalan, P. T. R. et al. Interaction of dihydrofolate reductase with methotrexate: ensemble and single-molecule kinetics. *Proc. Natl. Acad. Sci. USA* **99**, 13481–13486 (2002). 12359872 10.1073/pnas.172501499 PMC129699

[46] Singh, R. K. et al. Methotrexate disposition, anti-folate activity and efficacy in the collagen-induced arthritis mouse model. *Eur. J. Pharmacol.* **853**, 264–274 (2019). 30951714 10.1016/j.ejphar.2019.03.052 PMC6500488

[47] Wang, J. et al. Cardiomyocyte overexpression of miR-27b induces cardiac hypertrophy and dysfunction in mice. *Cell Res.* **22**, 516–527 (2012). 21844895 10.1038/cr.2011.132 PMC3292295

[48] Park, H. K. et al. Pseudohypoxic stabilization of HIF1alpha via cyclophilin D suppression promotes melanoma metastasis. *Signal Transduct. Target Ther.* **10**, 231 (2025). 40701956 10.1038/s41392-025-02314-8 PMC12287346

[49] Pellegrino, L. et al. miR-23b regulates cytoskeletal remodeling, motility and metastasis by directly targeting multiple transcripts. *Nucleic Acids Res.* **41**, 5400–5412 (2013). 23580553 10.1093/nar/gkt245 PMC3664824

[50] Cho, S. et al. miR-23∼27∼24 clusters control effector T cell differentiation and function. *J. Exp. Med.* **213**, 235–249 (2016). 26834155 10.1084/jem.20150990 PMC4749926

[51] Bang, C., Fiedler, J. A. N. & Thum, T. Cardiovascular Importance of the MicroRNA-23/27/24 Family. *Microcirculation* **19**, 208–214 (2012). 22136461 10.1111/j.1549-8719.2011.00153.x

[52] Chiang, H. R. et al. Mammalian microRNAs: experimental evaluation of novel and previously annotated genes. *Genes Dev.* **24**, 992–1009 (2010). 20413612 10.1101/gad.1884710 PMC2867214

[53] Lieber, D. S. et al. Targeted exome sequencing of suspected mitochondrial disorders. *Neurology* **80**, 1762–1770 (2013). 23596069 10.1212/WNL.0b013e3182918c40 PMC3719425

[54] El-Hattab, A. W. & Scaglia, F. Mitochondrial cardiomyopathies. *Front. Cardiovasc. Med.* **3**, 25 (2016). 27504452 10.3389/fcvm.2016.00025 PMC4958622

[55] Semsarian, C., Ingles, J., Maron, M. S. & Maron, B. J. New perspectives on the prevalence of hypertrophic cardiomyopathy. *J. Am. Coll. Cardiol.* **65**, 1249–1254 (2015). 25814232 10.1016/j.jacc.2015.01.019

[56] Bonow, R. O. & Greenland, P. Population-wide trends in aortic stenosis incidence and outcomes. *Circulation* **131**, 969–971 (2015). 25691712 10.1161/CIRCULATIONAHA.115.014846

[57] Takeda, A., Sakata, A. & Takeda, N. Image and DNA analysis of hypertrophic myocytes in hypertensive heart disease and hypertrophic cardiomyopathy. *Anal. Quant. Cytol. Histol.* **21**, 454–457 (1999). 10560530

[58] Zimmer, H. G., Trendelenburg, C. & Gerlach, E. Acceleration of adenine nucleotide synthesis de novo during development of cardiac hypertrophy. *J. Mol. Cell Cardiol.* **4**, 279–282 (1972). 4260708 10.1016/0022-2828(72)90065-x

[59] Wang, J. & Yang, X. The function of miRNA in cardiac hypertrophy. *Cell Mol. Life Sci.* **69**, 3561–3570 (2012). 22926414 10.1007/s00018-012-1126-y PMC3474911

[60] Nozato, T. et al. Overexpression of cdk Inhibitor p16INK4a by adenovirus vector inhibits cardiac hypertrophy in vitro and in vivo: a novel strategy for the gene therapy of cardiac hypertrophy. *J. Mol. Cell Cardiol.* **33**, 1493–1504 (2001). 11448137 10.1006/jmcc.2001.1412

[61] Tamamori, M. et al. Essential roles for G1 cyclin-dependent kinase activity in development of cardiomyocyte hypertrophy. *Am. J. Physiol.* **275**, H2036–H2040 (1998). 9843802 10.1152/ajpheart.1998.275.6.H2036

[62] Zhong, W. et al. Hypertrophic growth in cardiac myocytes is mediated by Myc through a Cyclin D2-dependent pathway. *EMBO J.* **25**, 3869–3879 (2006). 16902412 10.1038/sj.emboj.7601252 PMC1553193

[63] Ahuja, P. et al. Re-expression of proteins involved in cytokinesis during cardiac hypertrophy. *Exp. Cell Res.* **313**, 1270–1283 (2007). 17316608 10.1016/j.yexcr.2007.01.009

[64] Liu, Z., Yue, S., Chen, X., Kubin, T. & Braun, T. Regulation of cardiomyocyte polyploidy and multinucleation by CyclinG1. *Circ. Res.* **106**, 1498–1506 (2010). 20360255 10.1161/CIRCRESAHA.109.211888

[65] Frawley, L. E. & Orr-Weaver, T. L. Polyploidy. *Curr. Biol.* **25**, R353–R358 (2015). 25942544 10.1016/j.cub.2015.03.037

[66] Grove, D., Nair, K. G. & Zak, R. Biochemical correlates of cardiac hypertrophy. 3. Changes in DNA content; the relative contributions of polyploidy and mitotic activity. *Circ. Res.* **25**, 463–471 (1969). 4242254 10.1161/01.res.25.4.463

[67] Koda, M. et al. Nuclear hypertrophy reflects increased biosynthetic activities in myocytes of human hypertrophic hearts. *Circ. J.* **70**, 710–718 (2006). 16723792 10.1253/circj.70.710

[68] Matturri, L., Milei, J., Grana, D. R. & Lavezzi, A. M. Characterization of myocardial hypertrophy by DNA content, PCNA expression and apoptotic index. *Int. J. Cardiol.* **82**, 33–39 (2002). 11786155 10.1016/s0167-5273(01)00578-2

[69] Yabe, Y. & Abe, H. Changes in DNA synthesis in significantly hypertrophied human cardiac muscle. *Adv. Myocardiol.* **1**, 553–563 (1980). 7190317

[70] Derks, W. & Bergmann, O. Polyploidy in cardiomyocytes: roadblock to heart regeneration?. *Circ. Res.* **126**, 552–565 (2020). 32078450 10.1161/CIRCRESAHA.119.315408

[71] Mangoni, A. A., Zinellu, A., Sotgia, S., Carru, C. & Erre, G. L. Methotrexate and cardiovascular protection: current evidence and future directions. *Clin. Med. Insights: Ther.* **9**, 1179559X1774128 (2017).

[72] Ridker, P. M. et al. Low-dose methotrexate for the prevention of atherosclerotic events. *N. Engl. J. Med.* **380**, 752–762 (2019). 30415610 10.1056/NEJMoa1809798 PMC6587584

[73] Chen, J., Kubalak, S. W. & Chien, K. R. Ventricular muscle-restricted targeting of the RXRalpha gene reveals a non-cell-autonomous requirement in cardiac chamber morphogenesis. *Development* **125**, 1943–1949 (1998). 9550726 10.1242/dev.125.10.1943

[74] Ryan, H. E. et al. Hypoxia-inducible factor-1alpha is a positive factor in solid tumor growth. *Cancer Res.* **60**, 4010–4015 (2000). 10945599

[75] Haase, V. H., Glickman, J. N., Socolovsky, M. & Jaenisch, R. Vascular tumors in livers with targeted inactivation of the von Hippel-Lindau tumor suppressor. *Proc. Natl. Acad. Sci. USA* **98**, 1583–1588 (2001). 11171994 10.1073/pnas.98.4.1583 PMC29300

