# Supplementary material for: Hypoxia-driven microRNA-27b underlies pathologic cardiac endoreplication in heart disease
Source: Signal Transduct Target Ther. 2026 May 14;11:179. doi: 10.1038/s41392-026-02656-x (PMC13176343; doi:10.1038/s41392-026-02656-x)
Supplement: Supplementary file 1 — Supplementary Material [file 41392_2026_2656_MOESM1_ESM.pdf]

Supplementary Materials for

Hypoxia driven microRNA 27b underlies pathologic cardiac endoreplication in heart disease

Peter Mirtschink[1]†, Ting Yuan[2,3,5,6]†, Corinne Bischof[2,3,4]†, Minh Duc Pham[2,7], Chaonan Zhu[2,3,5,6], Akshay Ware[2], Yijie Mao[2,3,5], Meiqian Wu[2,3], Eva-Maria Rogg[2], Katharina Bottermann[2], Suam Gonzalez-Gonoggia[4], Corinne Berthonneche[8], Bettina Gercken[1], Eman Hagag[1], Katrin Strassburger[1], Samuel Sossalla[9,10], Sebastian N. Stehr[11], Wesley Abplanalp[2,5,6], Nicola Zamboni[12], Fabio Martelli[13], Thierry Pedrazzini[8,14], Markus Stoffel[15], Stefanie Dimmeler[2,5,6], and Jaya Krishnan[2,3,4,5,6]*

Correspondence to: Krishnan@med.uni-frankfurt.de

† Contributed equally

* Corresponding author

#: These authors contributed equally

This PDF file includes:

Materials and Methods
Supplementary Figures 1 to 9
Supplementary Table 1
Supplementary Table 2
Supplementary Table 3

Materials and Methods

Transverse aortic constriction (TAC)

9-14 week old mice were subjected to transverse aortic constriction (TAC) through constriction of the descending aorta as described [78]. The mice were monitored up to 9 weeks after surgery and their heart dimensions and functions were determined by echocardiography. *In vivo* miRCURY LNA Power Inhibitors were injected intraperitoneally (i.p.) into C57BL/6J mice at a dose of 10 mg/kg for 4 consecutive days at 49 days post-surgery as described in **Fig. 4a**. The following *In vivo* miRCURY LNA Power Inhibitors were purchased from Exiqon: i-mmu-miR-27b-5p (199900). i-Cel-control_inh (199900) was used as scrambled control LNA. In experiments with methotrexate versus placebo treatment, methotrexate or placebo were injected i.p. at a dose of 1.5mg/kg/week for for 4 consecutive weeks as described in **Fig. 6C**.

In vivo transthoracic ultrasound imaging

Transthoracic echocardiography was performed using the MS400 (18-38MHz) probe from Vevo 2100 color doppler ultrasound machine (VisualSonics). Mice were lightly anesthetized with 1-1.5% isoflurane, maintaining heart rate at 400-550 beats per minute. The mice were placed in decubitus dorsal on a heated 37ºC platform to maintain body temperature. A topical depilatory agent is used to remove the hair and ultrasound gel is used as a coupling medium between the transducer and the skin. Hearts were imaged in the 2D mode in the parasternal long-axis view. From this view, an M-mode cursor was positioned perpendicular to the inter-ventricular septum and the posterior wall of the left ventricle at the level of the papillary muscles. Diastolic and systolic interventricular septum diameter (IVS;d and IVS;s), diastolic and systolic left ventricular posterior wall diameter (LVPW;d and LVPW;s), and left ventricular internal end-diastolic and end-systolic chamber (LVID;d and LVID;s) dimensions were measured. The measurements were taken in three separate M-mode images and averaged. Left ventricular fractional shortening (%FS) and ejection fraction (%EF) was also calculated. Fractional shortening was assessed from M-mode based on the percentage changes of left ventricular end-diastolic and end-systolic diameters. %EF is derived from the formula of (LV vol;d – LV vol;s)/ LV vol;d×100. At the end of the duration of the experiment, the animals were sacrificed and the heart weight-to-body weight ratio was measured.

Isolation and maintenance of primary neonatal rat cardiomyocytes

Isolation of primary neonatal rat cardiomyocytes (NRC) was performed using the neonatal heart dissociation kit (130-098-373, Miltenyi Biotec) as recommended by the manufacturer. Isolated cells were pre-plated with plating medium (65% DMEM, 16% M199, 10% fetal calf serum (FCS), 5% horse serum (HS), 2% glutamine and 1% penicillin/streptomycin (P/S)) for 1.5 h to deplete of the fibroblasts. NRC were plated on

Type-I Collagen-coated (Advanced Biomatrix) 3 cm dishes (Nunc) in plating medium. The plating medium was changed to maintenance medium (88% DMEM, 9% M199, 1% HS, 2% glutamine and 1% P/S) 24 h after isolation of NRCs. Cardiomyocytes were treated with Triiodothyronine (T3, T5516, Sigma Aldrich) for 6 days at a concentration of 15 nM.

Isolation of adult mouse cardiomyocytes
The left ventricle of adult mouse hearts was cut into pieces of 1 mm^3 and fixed for 2 h with 4% paraformaldehyde (PFA)/PBS. The biopsies were digested with 1000 U collagenase type II (17101-015, Gibco) in HBSS for approximately 42 h at 37°C while rotating. Appropriate concentrations of isolated adult cardiomyocytes were centrifuged at 600 rpm for 1 min onto gelatine-coated microscope slides using a cytospin (SCA-0030, Shandon Southern).

Lentivirus production and infection
Mycoplasma-free authenticated HEK-293T (ATCC) cells were transfected at 80-90% confluence with polyethylenimine (PEI) transfection reagent. 10 µg transgene, 7.5 µg pMD2.G and 6.5 µg psPAX2 were mixed with 2 ml serum-free DMEM and 45 µg PEI per 10 cm dish. After 10 min incubation at room temperature (RT) the DNA/PEI-complexes were added slowly to the cells cultured in DMEM containing 0.5% FCS and L-glutamine. Medium was changed to NRC maintenance medium 4 h after transfection. Lentiviruses were harvested 48 h after transfection and stored at -80°C. NRC were infected 20 h after isolation and incubated at 37°C/5% CO_2 overnight.

AAV9 constructs
AAV9-loxP-STOPcassette-loxP-miR-27b were from Vector Biolabs and were injected at a concentration of $1.2x10^{13}$ genome copies (GC) per kg body weight into tail veins of 3-week-old *Mlc2v-cre*$^-$ and *Mlc2v-cre*$^+$ mice.

In vitro administration of locked nucleic acids (LNA), miRNA mimics and siRNAs
miRCURY LNA Power Inhibitors were added directly to the cell culture medium at a final concentration of 50 nM and fresh LNAs added every second day. The following miRCURY LNA Power Inhibitors were purchased from Exiqon: i-mmu-miR-27b-5p (4101712-101). Negative Control A (199006-101) was used as non-targeting LNA.
miRIDIAN microRNA Mimics were transfected into NRCs using Lipofectamine 2000 (Invitrogen). Per well in a 96-well plate, 0.4 µL Lipofectamine 2000 was mixed with 25 µL OptiMEM (Invitrogen). After incubation of 5 min at RT, the mixture was added to an Eppendorf tube containing 6.25 nM or 12.5 nM miRNA mimics in 50 µL OptiMEM and incubated for 20 min at RT to form the complexes. 50 µL of the complexes was added to the cells cultured in 100 µL medium without antibiotics. The medium was replaced 4 h after transfection and cells were harvested 48 h after transfection. The following

miRIDIAN microRNA Mimics were purchased from Dharmacon: mmu-miR-27b-3p (C-310380-05-0005), mmu-miR-27b-5p (C-310810-01-0005). miRIDIAN microRNA Mimic Negative Control #1 (CN-001000-01) was used as negative control.

Plasmid constructions

The pLenti-HIF1α puro lentiviral expression vector was generated by subcloning the HIF1α fragment from a pcDNA3-HA-HIF1α(401–603) plasmid [23] into pLenti-pgk puro vector as described previously [79]. As a corresponding control the pLenti-pgk puro empty vector was used. The miR27b overexpression construct was generated by amplification of the precursor mir27b sequence and the flanking sequence of 158 bp from either end of the mir27b precursor transcript from mouse genomic DNA. The sequence was cloned into the pLKO.1 CMV puro construct provided by A. Ittner (ETH Zurich), by using EcoRI and SalI restriction sites. As a control vector the empty pLKO.1 CMV puro construct was used.

Generation of miRNA Promoter-Luciferase Constructs

A 1.0 kilobyte (kb) fragment of the miR-23b, miR-24-1 and miR-27b promoter was amplified from mouse genomic DNA and cloned into the pGL3 luciferase reporter vector (Stratagene) between the XhoI and HindIII restriction sites. Mutation of the HRE in the mir27b promoter was generated by recombinant PCR [80]. Sense and Antisense primers were designed bearing HRE mutations, which were used to amplify the mutant 5' and 3' regions of the miR-27b promoter, respectively. Afterwards, the 5' and 3' products generated from the respective PCR reactions were mixed at a 1:1 ratio and the entire fragment was amplified using primers targeting the 5' and 3' ends of the promoters. The wildtype constructs and the mutation of the HRE in the miR-27b promoter were confirmed by DNA sequencing (Microsynth).

Generation of Atp5a1 3' UTR Luciferase Construct

3' UTR of *Atp5a1* was amplified from mouse cDNA and cloned into the pmirGLO Dual-Luciferase miRNA Target Expression Vector (Promega). Mutation of miR-27b binding site on the 3' UTR of *Atp5a1* was generated by recombinant PCR [81,82]. Sense and antisense primers were generated bearing miR-27b binding site mutations, which were used to amplify the mutant 5' and 3' regions of the 3'UTR, respectively. Afterwards, the 5' and 3' products generated from the respective PCR reactions were mixed at a 1:1 ratio and the entire sequence was amplified using primers targeting the 5' and 3' ends of the *Atp5a1* 3'UTR. The mutation of the miR-27b binding site in the 3' UTR of *Atp5a1* was confirmed by sequencing (Microsynth).

Lentiviral and expression constructs

For knockdowns with lentiviral shRNAs the following TRC pLKO.1 shRNA vectors were

used: Atp5a1 (shAtp5a1, TRCN0000076239), Hif1α (shHif1α, TRCN0000232220) and Vhl (shVhl, TRCN0000436052). pLKO.1 vector containing non-silencing shRNA (nsRNA; SHC002, Sigma) was used as a control. pLenti ATP5A1 (RC214840L1) plasmid was from Origene.

RNA-isolation, reverse transcription and qRT-PCR

Samples were harvested in Trizol (Invitrogen) and total RNA isolated as recommended by the manufacturer. 750 ng RNA were reverse transcribed into cDNA using RNA to cDNA EcoDry Premix (random hexamers) kit (Clontech, Cat No 639545) following the manufacturer's instructions. Quantitative real-time PCR (qRT-PCR) reactions were set up using iTaq Universal SYBR Green Supermix (Biorad, Cat No 1725121) according to manufacturer's recommendations and run on a PikoReal Real-Time PCR machine (Thermo Scientific). *C*t values were normalized against the housekeeping gene *Hprt1*. The qRT-PCR primer sequences are shown in **Supplementary Table 3**.

To assess mature miRNA levels, 10 ng total RNA was transcribed into cDNA using TaqMan MicroRNA Reverse Transcription Kit (4366596, Applied Biosystems) as recommended by the manufacturer. qRT-PCR was performed using TaqMan 2x Universal PCR Master Mix (4304437, Applied Biosystems) following the manufacturer's instructions and run on a DNA Engine Opticon 2 (Bio-Rad). *C*t values were normalized against the housekeeping genes *snoRNA* (rat) or *snoRNA202* (mouse). The following primers from Thermo Fisher Scientific were used: miR-23b-3p (ID000400), miR-23b-5p (ID243680_mat) miR-24-3p (ID000402), miR-24-5p (ID 000488), miR-27b-3p (ID000409), miR-27b-5p (ID002174), snoRNA (ID001718) and snoRNA202 (ID001232).

RNA Sequencing and Analysis

RNA isolation was performed using RNeasy Plus Micro Kit (QIAGEN) and RNA was subjected to the amplification workflow of the SMARTer Ultra HV v2 kit (Takara Bio). Amplified cDNA was successively converted into short read sequencing libraries using the NEBnext Ultra DNA library preparation chemistry (New England Biolabs). Libraries were equimolarly pooled and sequenced on an Illumina HiSeq 2500, resulting in ~50 million single end reads per library.

FastQC (http://www.bioinformatics.babraham.ac.uk/) was used to perform a basic quality control on the resulting reads. As an additional control, library diversity was assessed by redundancy investigation in the reads. Alignment of the reads to the rat reference assembly, mRatBN7.2 was done with GSNAP. Normalization of the raw read counts based on the library size and testing for differential expression between conditions was performed with the DESeq2 R package [83]. Genes, which have an adjusted *p* value (padj) < 0.05 and counts > 50 were considered as differentially expressed. To perform gene set enrichment analysis (GSEA), gene sets were ranked by taking the -log10 transform of the p value and signed as positive or negative based on the direction of fold

change. GSEA pre-ranked analysis (1000 permutations, minimum term size of 15, maximum term size of 500) was then performed using the GSEA software (Broad Institute) [84]. Annotated gene sets from Molecular Signatures Database (MSigDB) were used as input. Lineage specific genes were selected based on IPA analysis (Molecular and Cellular Functions analysis). For generation of heatmaps of genes involved in folate metabolism, the synthesis of purine containing compounds, glycolysis, AMPK signaling, and hypertrophic cardiomyopathy we used the GO, Hallmark and Wikipathways databases.

Chromatin immunoprecipitation

ChIP assays were performed with material from NRCs and the assays carried out using the ChIP-IT Kit (Active Motif) according to the manufacturer's instructions and analyzed by qRT-PCR. ChIP was performed with a ChIP-grade antibody against Hif1α (mouse, ab1, Abcam). *In silico* promoter analyses and alignments were performed using MatInspector and DiAlignTF (Genomatix). Primer sequences used for miR-27b in the ChIP were 5′-GCATGCTGATTTGTGACTTGAG-3' and 5′-CCTCTGTTCTCCAAACTGCAG-3′.

MicroRNA microarray

The microRNA microarrays were performed on 3 biological replicates of *Vhl* cKO mice and three control mice (*Vhl* fl/fl), and on 3 biological replicates of *Hif1α* cKO mice subjected to TAC and three controls subjected to TAC surgery (TAC *Hif1α* fl/fl), respectively. Cardiac dimensions and function were confirmed in all mice by echocardiography. Total RNA was isolated from left ventricle and miRNAs were labelled using the miRCURY LNA microRNA Power Labelling Kit (Exiqon) and hybridized on miRNA arrays (miRXplore) that carry 1194 DNA oligonucleotides with the reverse-complementary sequence of the mature miRNAs. These arrays cover 728 human, 584 mouse, 426 rat and 122 viral miRNAs, each spotted on the arrays in quadruplicate. The Cy5-labelled miRNAs were normalized to a reference pool of miRNAs that were simultaneously labeled with Cy3. All the data are represented as ratios of logarithmic values between the diseased and control animals and deposited under GSE62418.

ATP quantification in vivo

ATP was separated and quantified on an anion exchange column (Nucleosil 4000-7 PEI, 50/4 from Macherey-Nagel) with a linear gradient (0–1.5 M NaCl in 10 mM Tris-HCl, pH 8.0) using an HPLC system equipped with two independent UV-visible spectrometers (Shimadzu,). Elution of samples was monitored at 259 and 220 nm. The 220 nm wavelength was used to detect possible traces of contaminants.

ADP and ATP assays

For the ADP/ATP ratio analysis, 4x10^5 NRCs were seeded per well in a 3cm dish and the assay was performed using the EnzyLight ADP/ATP Ratio Assay Kit (ELDT-100, Bioassay Systems) as recommended by the manufacturer. For ADP and ATP quantifications the EnzyLight™ ADP Assay Kit (EADP-100, BioAssay Systems) and EnzyLight ATP Assay Kit (EATP-100, BioAssay Systems) were used as recommended by the manufacturers, respectively. The signal was measured on a FLUOstar Omega plate reader (BMG). For ADP/ATP quantification in human and mouse heart tissue, the assay was performed using the ADP/ATP Ratio Assay Kit (ab65313, Abcam) as recommended by the manufacturer with small modifications. Briefly, the heart tissue was frozen and stored in liquid nitrogen immediately after the harvest. The tissue was powdered with a mortar and suspended in lysis buffer (10 µl/mg of tissue powder) for 5 min at room temperature. After the centrifugation at 10000 g for 1 min, the supernatant was used for the assay. Data was normalized to protein amount in the supernatant by the Bradford assay.

ATP synthase Enzyme Activity Assay
4x10^5 cells were seeded per 3 cm dish. Cells were harvested by trypsinization, followed by centrifugation at 1.2 krpm for 5 min. Pellet was washed 1x with PBS (Invitrogen) and centrifuged at 1.2 krpm for 5 min. Pellet was resuspended in 100 µl PBS and frozen at -80°C and sample preparation was continued using the ATP synthase Enzyme Activity Assay Kit (ab109714, Abcam) as recommended by the manufacturer. Absorbance was measured at 340 nm and 30°C for 3 h at 1 min intervals using a FLUOstar Omega plate reader (BMG Labtech). ATP synthase activity was normalized to protein concentration.

Luciferase assay
4x10^4 NRCs were plated in a white 96-well plate and cultured for 3 days. 40 ng of wildtype or mutant pmirGLO Atp5a1 3'UTR construct was co-transfected with 6.25, 12.5 or 25 nM control or miR27b mimics using Lipofectamine 2000 (Invitrogen) as recommended by the manufacturer. Luciferase activity was measured 24 h after transfection using the Dual Luciferase Reporter Assay System (Promega) as recommended by the manufacturer on a FLUOstar Omega Microplate Reader (BMG Labtech).
To assess promoter activity, 20 ng pGL3 vector was co-transfected with 1.25 ng *Renilla* and 0-260 ng HIF1αΔODD. Luciferase activity was measured 36 h after transfection using the Dual Luciferase Reporter Assay System (Promega) as recommended by the manufacturer. E2F transactivation activities were measured using the Cignal Reporter Assay Kit (CCS-003L, Qiagen) according to the manufacturer's instructions using the Dual Luciferase Reporter Assay System (Promega).

Immunoblotting
Heart tissue was solubilized in blue wonder sample buffer (3.7 M urea, 134.6 mM Tris pH 6.8, 5.4% (v/v) sodium dodecyl sulphate (SDS), 2.3% (v/v) NP-40, 4.45% (v/v) β-

mercapto-ethanol, 4% (v/v) glycerol, 60 mg/L bromophenol blue) and proteins denatured for 5 min at 95°C after homogenization using an Ultra-Turrax T10 tissue homogeniser (IKA). NRCs were washed twice with ice-cold PBS and harvested in blue wonder sample buffer. Samples were sonicated to reduce viscosity using a Bransonic 5510 Ultrasonic water bath (Branson) and boiled for 5 min. After brief centrifugation, protein lysates were loaded into 8% or 10% polyacrylamide minigels (Biorad) and transferred to nitrocellulose membrane (GE Healthcare) by wet transfer. Membranes were blocked in 5% (w/v) milk powder (Biorad) in TBST before incubation with primary antibody diluted in 5% (w/v) bovine serum albumin (BSA) in TBST for 2 hours at room temperature or overnight at 4°C. Following three washes in 5% (w/v) milk powder in TBST, membranes were incubated for 1-2 hours with the appropriate HRP conjugated secondary antibody (anti-Goat IgG HRP, 61-1620; anti-Mouse IgG HRP, 62-6520; anti-Rabbit IgG HRP, 65-6120, Invitrogen) at a dilution of 1:5000. Membranes were then washed three times with TBST before detection with ECL (Amersham) on X-ray RX NIF films (Fisher Scientific) to detect the chemiluminescence. Signal intensities were quantified by densitometry using Image J (version 1.47) [85]. The following antibodies were used for immunoblotting: anti-α-actinin (A7811, Sigma), anti-Atp5a1 (ab14748, Abcam), anti-cardiac actin (61075, Progen Biotechnik), anti-Hif1α (NB100-479, Novus Biologicals) and anti-Vhl (2738, Cell Signaling).

Immunofluorescence stainings

Immunofluorescent stainings were performed as described previously [6]. After fixation of NRCs with 4% PFA/PBS, the cells were permeabilized and incubated with primary antibodies diluted in 2% (v/v) HS for 1 h at RT. After 3 washes with PBS for 5 min, cells were incubated with 4′,6-diamidino-2-phenylindole (DAPI; D1306, Thermo Fisher Scientific, 0.1 µg/ml), Phalloidin 555 (A34055, Molecular Probes), AlexaFluor 647 anti-mouse (A-11001, Thermo Fisher Scientific) and AlexaFluor 488 anti-mouse (A-21235, Thermo Fisher Scientific) secondary antibody for 1 h at room temperature. For adult cardiomyocytes, the slides were incubated with the primary and secondary antibodies overnight at 4°C in a humidified chamber. Slides and dishes were mounted onto glass slides (Fisher Scientific) with a drop of ProLong Antifade (Thermo Fisher Scientific). The slides/dishes were fixed with clear nail polish and left to dry. The following primary antibodies were used: sarcomeric α-actinin (A7811, Sigma Aldrich), Ki67 (ab15580, Abcam) and phospho-Histone H3 (Ser10) (05-817, Cell Signaling). Fluorescent images were acquired with the SP5 confocal microscopy (Leica) using a 20x magnification. Cell size was quantified blinded using the software Image J (version 1.47).

Immunohistochemistry

Hearts were embedded in optimal cutting temperature (OCT) compound and sectioned at 10 µm. Sections were fixed for 10 min with 4% PFA/PBS and after 2 washes with PBS for

2 min blocked for 1 h with 2% HS/PBS for 1 h at room temperature. After permeabilisation for 10 min with 0.2% Triton X-100/PBS, the sections were washed 3 times with PBS for 5 min and incubated with primary antibodies against sarcomeric α-actinin (A7811, Sigma Aldrich, 1:800) and Laminin (ab11575, Abcam, 1:300) diluted in 2% (v/v) HS overnight in a humidified chamber at 4°C. After 3 washes with PBS for 10 min, sections were incubated with 4′,6-diamidino-2-phenylindole (DAPI; D1306, Thermo Fisher Scientific, 0.1 µg/ml), AlexaFluor 555 anti-mouse (A-21422, Thermo Fisher Scientific, 1:500) and AlexaFluor 488 anti-rabbit (A-11034, Thermo Fisher Scientific, 1:500) secondary antibody for 2 h at room temperature. Sections mounted with a drop of ProLong Antifade (Thermo Fisher Scientific) and fixed with clear nail polish and left to dry. Fluorescent images were acquired with the SP8 confocal microscopy (Leica) using a 20x magnification. The entire z-axis was imaged and z-stack image generated from approximately 0.2 µm steps. To determine the number of nuclei per myocyte we included only myocytes that had been cut along their longitudinal axis. Nuclei that were surrounded by the extracellular matrix stain laminin were excluded from the analysis. Quantification of nuclei was performed blinded. Cryosections were stained with hematoxylin and eosin (H&E) or picrosirius red (Sigma). Slides were visualized using a Motic AE2000 with a Moticam 3 or the Axioscan.Z1 slide scanner (Carl Zeiss).

[^{3}H]leucine incorporation assay

[^{3}H]leucine incorporation assay was used to measure *de novo* protein synthesis as an indirect readout for cell growth [86]. 4x10^5 cells were seeded per 3 cm dish. After 3 days, cells were serum-starved overnight. Next day, cells were cultured in leucine-free medium for 4h, followed by culturing in maintenance medium containing labeled L-[4,5-^{3}H(N)]isoleucine (specific activity 30-60 Ci/mmol, ART0233, American Radiolabelled Chemicals) at a concentration of 0.5 µCi/mL for 20 h. The next day, cells were washed with PBS, trypsinized and the radioactivity was measured for 5 min in in the liquid scintillation analyzer Tri-Carb 2800TR (Perkin Elmer). Cells were counted blinded with a Neubauer chamber after trypsinization and scintillation counts were normalized to absolute cell number.

Nucleic acid incorporation assays

4x10^5 cells were seeded per 3 cm dish. After 4 days, cells were starved of glucose for 1.5 h and serine or glycine for 3 h. For radiolabelled glucose incorporation, cells were incubated for 4 h in glucose-free DMEM (Invitrogen) containing 1 µCi/mL uniformly labeled [U-^{14}C]glucose (specific activity 250-360 mCi/mmol, NEC042V250UC, Perkin Elmer). For radiolabelled serine incorporation, cardiomyocytes were incubated overnight in MEM (Invitrogen) supplemented with MEM Vitamin Solution (Invitrogen) and containing 0.6 µCi/mL radiolabelled serine at carbon 3 ([3-^{14}C]serine, specific activity 50-62 mCi/mmol, NEC827050UC, Perkin Elmer). For radiolabelled glycine incorporation,

cells were incubated in BME (Invitrogen) containing 1 μCi/mL uniformly radiolabelled glycine ([^{14}C(U)]glycine, specific activity >100mCi/mmol, NEC276E250UC, Perkin Elmer) for 4 h. After washing the cells three times with PBS, cells were harvested in Trizol (Invitrogen) and nucleic acids isolated as recommended by the manufacturer. Isolated nucleic acids were transferred to a scintillation vial containing 4 mL of the liquid scintillation cocktail Ultima Gold (Perkin Elmer) and radioactivity was measured for 5 min in the Liquid Scintillation Analyzer Tri-Carb 2800TR (Perkin Elmer). Scintillation counts were normalized to absolute nucleic acid quantity.

Fatty acid oxidation

Fatty acid oxidation rate in animal tissues using [1-^{14}C]-palmitic acid (NEC075H, Perkin Elmer) was determined as described previously [87] with the exception that 0.7% BSA/500 μM palmitate/2 μCi ^{14}C-palmitate was used per reaction. Radioactivity was measured for 5 min in the Liquid Scintillation Analyzer Tri-Carb 2800TR (Perkin Elmer). Scintillation counts were normalized to protein amount.

Flow cytometry

1x10^6 NRCs were plated on a 6 cm dish (Nunc) and after 4-6 days cells were harvested by trypsinization. Cells were washed 1x with PBS and centrifuged at 80 g for 5 min. Cells were resuspended in 500 μL PBS and 5 mL cold 70% (v/v) ethanol (kept at -20°C) was added immediately. The fixed cells were kept at 4°C for up to 1 week. Prior to flow cytometry analysis, cells were centrifuged and washed with PBS. After centrifugation at 80 g for 5 min the cells were resuspended in 500 μL propidium iodide solution (69 μM propidium iodide in 38 mM sodium citrate, pH 7.4) containing 40 μg/mL RNase and incubated for 1 h at 37°C. Samples were run on the ImageStream (Amnis), a flow cytometer coupled to fluorescence image acquisition to obtain representative images of the flow cytometry data. The multinucleation was quantified from the ImageStream images.

Formate assay

4x10^5 cells were seeded per 3 cm dish and the assay was performed using the Formate Assay Kit (Sigma). Cells were washed twice with cold PBS and collected in 25 μL Formate Assay Buffer by scraping. After centrifugation at 15000 g at 4°C for 5 min, 20 μL lysate was mixed with 5 μL Formate Assay Buffer and added to a 96-well plate. 25 μL of the Reaction Mix consisting of 23 μL Formate Assay Buffer, 1 μL Formate Enzyme Mix and 1 μL Formate Substrate Mix was added to the plate containing the lysate. The samples were incubated for 1 h at 37°C protected from light and the absorbance was measured at 450 nm on a FLUOstar Omega plate reader (BMG Labtech). The data was normalized to protein concentration.

Metabolomics

4×10^5 NRCs were cultured per 3 cm dish. The whole cell culture plates were snap frozen in liquid nitrogen after the cells were washed with 75 mM Ammonium carbonate (Sigma), adjusted to pH 7.4 with acetic acid. The metabolites were extracted with cold extraction buffer (–20°C) containing acetonitrile:methanol:water in a 40:40:20 ratio. Untargeted analysis of metabolites by flow injection–time-of-flight mass spectrometry as previously described [88]. Data was processed and analyzed with Matlab. Metabolomics analysis performed by Metabolon (**Supplementary Fig. 5d**) was performed as previously described [89]. Morpheus software (Broad Institute) was used to generate heatmaps.

Lipidomics

Mouse heart samples were homogenized on ice in ammonium-bicarbonate buffer (150 mM ammonium bicarbonate, pH 7) with ultra-turax homogenizer. Protein content was assessed using BCA Protein Assay Kit (Thermo Fisher). Equivalents of 20 µg of protein were taken for mass spectrometry analysis. Mass spectrometry-based lipid analysis was performed by Lipotype GmbH (Dresden, Germany) as described [90]. Lipids were extracted using a two-step chloroform/methanol procedure [91]. Samples were spiked with internal lipid standard mixture containing: cardiolipin 16:1/15:0/15:0/15:0 (CL), ceramide 18:1;2/17:0 (Cer),, hexosylceramide 18:1;2/12:0 (HexCer), lyso-phosphatidate 17:0 (LPA), lyso-phosphatidylcholine 12:0 (LPC), lyso-phosphatidylethanolamine 17:1 (LPE), lyso-phosphatidylglycerol 17:1 (LPG), lyso-phosphatidylinositol 17:1 (LPI), lyso-phosphatidylserine 17:1 (LPS), phosphatidate 17:0/17:0 (PA), phosphatidylcholine 17:0/17:0 (PC), phosphatidylethanolamine 17:0/17:0 (PE), phosphatidylglycerol 17:0/17:0 (PG), phosphatidylinositol 16:0/16:0 (PI), phosphatidylserine 17:0/17:0 (PS), cholesterol ester 20:0 (CE), sphingomyelin 18:1;2/12:0;0 (SM), cholesterol D6 (Chol) (all Avanti Polar Lipids), triacylglycerol 17:0/17:0/17:0 (TAG) and diacylglycerol 17:0/17:0 (DAG) (both Larodan Fine Chemicals). After extraction, the organic phase was transferred to an infusion plate and dried in a speed vacuum concentrator. 1st step dry extract was re-suspended in 7.5 mM ammonium acetate (Sigma) in chloroform/methanol/propanol (1:2:4, V:V:V) and 2nd step dry extract in 33% ethanol solution of methylamine in chloroform/methanol (0.003:5:1; V:V:V) (all liquids were LC grade obtained from VWR). All liquid handling steps were performed using Hamilton Robotics STARlet robotic platform with the Anti Droplet Control feature for organic solvents pipetting.

Samples were analyzed by direct infusion on a QExactive mass spectrometer (Thermo Scientific) equipped with a TriVersa NanoMate ion source (Advion Biosciences). Samples were analyzed in both positive and negative ion modes with a resolution of Rm/z=200=280000 for MS and Rm/z=200=17500 for MSMS experiments, in a single acquisition. MSMS was triggered by an inclusion list encompassing corresponding MS mass ranges scanned in 1 Da increments [92]. Both MS and MSMS data were combined to monitor CE, DAG and TAG ions as ammonium adducts; PC, PC O-, as acetate adducts; and CL, PA, PE, PE O-, PG, PI and PS as deprotonated anions. MS only was used to

monitor LPA, LPE, LPE O-, LPI and LPS as deprotonated anions; Cer, HexCer, SM, LPC and LPC O- as acetate adduct and cholesterol as ammonium adduct of an acetylated derivative [93].
Data were analyzed with a lipid identification software based on LipidXplorer [94,95]. Data post-processing and normalization were performed using an in-house developed data management system. Only lipid identifications with a signal-to-noise ratio >5, and a signal intensity 5-fold higher than in corresponding blank samples were considered for further data analysis.
All downstream analyses were performed in R [96]on the mol%-transformed dataset, i.e., after transforming raw data (picomol) to mole percent (each quantity was divided by the sum of the lipids detected in its respective sample and multiplied by 100). Principal Component Analysis (PCA) was computed using the Singular Value Decomposition function. Total Carbon chain length and saturation plots result from grouping together all the lipids that present the same number of carbon atoms (total length) or the same number of double bonds (saturation) and calculating their mean and standard deviation in each cohort of samples. The difference between the means was calculated for each species by subtracting the mean of the controls from the mean of the treated samples (Treated *minus* Reference), but only the lipids with a delta exceeding |0.3| are shown in the supplemental material. Significance was calculated by means of the non-parametric test Wilcoxon and, in case of multiple comparisons, p-values were adjusted after the Benjamini-Hochberg correction. Significance was set at $p<0.05$. Alongside R-base functions the following packages were used: factoextra [97], reshape2 [98] and ggplot2 [99]).

Bioinformatic analysis
In silico promoter analyses were performed using MatInspector (Genomatix). Sequence alignments were performed with BLAST alignment (http://www.ncbi.nlm.nih.gov/blast). shRNAs were designed by proprietary algorithm (Targeted Transgenesis), or computationally predicted using pSico Oligomaker (MIT, version 1.5), BLOCK-iT RNAi Designer (Life Technologies). To predict miR27b targets the miRWalk 2.0 database (http://mirwalk.uni-hd.de) which includes many databases such as TargetScan, miRanda and RNA22 was used[100]. Confocal images were quantified using Image J (version 1.47)[85].

Statistical analysis
Statistical analyses were performed using two-tailed unpaired Student's *t*-tests, or one-way or two-way ANOVA analyses followed by Tukey's multiple comparison post-test as indicated in the respective figure legends. For statistical analysis of survival curves, we performed log-rank (Mantel-Cox) test. A *P* value of less than 0.05 was considered statistically significant. No statistical methods were used to predetermine sample size.

Supplementary Figure 1

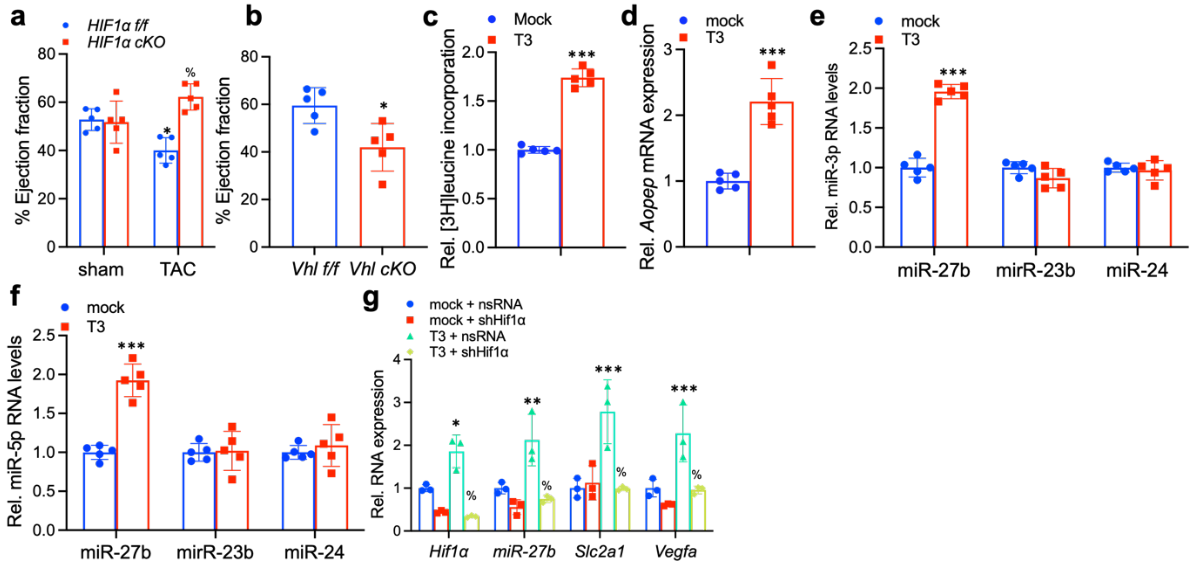

Supplementary Fig. 1 │ T3 induces miR-27b expression and function

a, b, Quantification of ATP amount in left ventricular biopsies from control (*Hif1α fl/fl*) and ventricle-specific *Hif1α* conditional knockout (*Hif1α cKO*) mice subjected to sham or TAC surgery (**a**), and from ventricle-specific *Vhl* conditional knockout (*Vhl cKO*) and respective control (*Vhl fl/fl)* mice (**b**). (n=5 mice per group for (**a**) and (**b**); shown is mean ± SD; *, % $P < 0.05$; two-way ANOVA (**A**) or two-tailed unpaired t-test (**b**)). **c,** Evaluation of [^{3}H]leucine incorporation in NRCs treated with T3 or PBS (mock). Data is represented as incorporated radioactivity relative to mock-treated NRCs (set as 1.0). (n=5 biological replicates per group; results shown are the mean ± SD; *** $P < 0.001$; two-tailed unpaired t-test). **d-f,** Relative mRNA expression of *Aopep* (**d**), mature miR-27b-3p, miR-23b-3p and miR-24-3p (**e**), and miR-27b-5p, miR-23b-5p and miR-24-5p (**f**) in NRCs treated with PBS (mock) or T3. Data is normalized to mock-treated NRCs (set as 1.0). n=5 biological replicates per group; shown is mean ± SD; *** p < 0.001; two-tailed unpaired t-test. **g,** Relative expression of *Hif1α*, *miR-27b*, *facilitated glucose transporter member 1 (Slc2a1/Glut1)* and *Vegfa* mRNA in NRCs stimulated with T3 and transduced with non-silencing control shRNA (nsRNA) or shRNA against Hif1α (shHif1α). Data is normalized to control NRCs infected with nsRNA (set as 1.0). n=3 biological replicates per group; shown is mean ± SD; * $P < 0.05$, ** $P < 0.01$, *** $P < 0.01$ vs. mock + nsRNA, % $P < 0.05$ vs. T3 + nsRNA; two-way ANOVA and Tukey's post-test.

Supplementary Figure 2

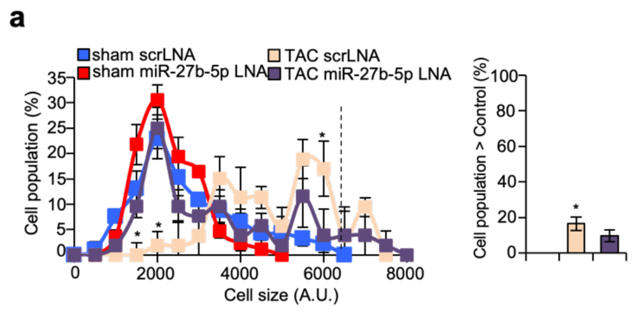

Supplementary Fig. 2 │ miR-27b drives pathological cell growth

a, Cardiomyocytes were isolated from sham or TAC mice treated with LNAs targeting miR27b-5p were stained for DAPI and α-actinin, and cell size distribution of cardiomyocytes from the respective groups quantified. Cells were imaged by confocal microscopy and a representative z-stack image from 3 mice per group is shown. Scale bar is 20 µm. n= 120-200 cardiomyocytes were analyzed per heart. * *P* < 0.05; two-tailed unpaired t-test.

Supplementary Figure 3

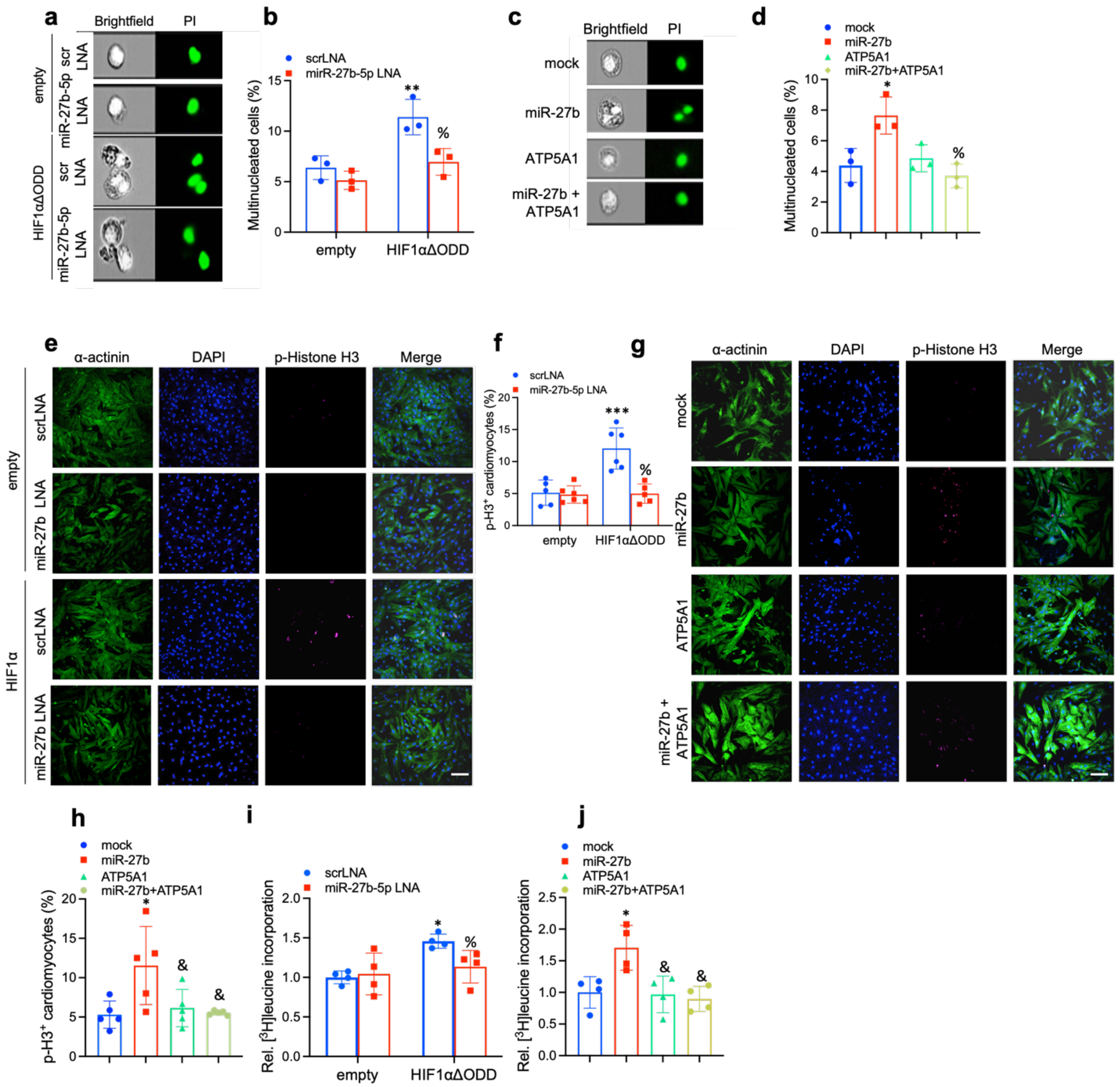

Supplementary Fig. 3 | HIF1α-miR-27b activation controls cardiomyocyte endomitosis and pathological growth

a-d, NRCs transduced and treated as indicated were stained with propidium iodide (PI) and assessed for polyploidy by flow cytometry coupled to imaging (**a, c**) and multinucleation quantified from images (**b, d**). (n=3 biological replicates per group with at least 200 cells quantified; results shown are the mean ± SD; ** $P < 0.01$, * $P < 0.05$; two-way ANOVA followed by Tukey's post-test (B); One-way ANOVA followed by Tukey's post-test (D)). **e-h,** NRCs transduced with the indicated lentiviruses were stained for the cardiac-specific marker α-actinin, DAPI and phospho-Histone H3 (p-Histone H3) for assessment of cell mitosis and imaged by

confocal microscopy. p-Histone H3 positive cardiomyocytes were quantified. 5-6 biological replicates with 3-4 fields/replicate were analyzed per condition with representative fields shown. *** $P < 0.001$ compared to scrLNA, % $P < 0.001$ vs. compared to scrLNA + HIF1αΔODD ; Two-way ANOVA followed by Tukey's post-test (**f**), *** $P < 0.05$ compared to mock control, & $P < 0.05$ vs. compared to miR-27b, one-way ANOVA followed by by Tukey's post-test (**h**). Scale bar is 100 μm. **i, j,** Evaluation of [^{3}H]leucine incorporation in NRCs transduced and treated as indicated. Data is represented as incorporated radioactivity relative to control NRCs (set as 1.0). (n=4 biological replicates per group; results shown are the mean ± SD; * $P < 0.05$ compared to scrLNA, % $P < 0.05$ compared to scrLNA + HIF1αΔODD; Two-way ANOVA followed by Tukey's post-test (I); * $P < 0.05$ compared to mock control; % $P < 0.05$ compared to miR-27b; One-way ANOVA followed by Tukey's post-test (**j**).

Supplementary Figure 4

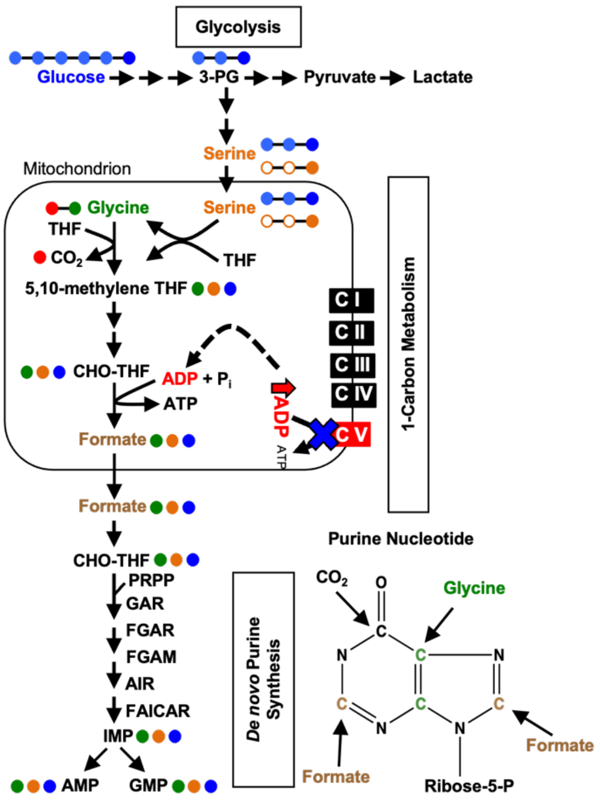

Supplementary Fig. 4 | Cellular carbon flux driving nucleotide biosynthesis

Schematic representation of *de novo* purine biosynthesis pathway showing the contribution of glycolysis and 1-carbon metabolism. The origin of the carbon atoms of the purine ring is indicated. Circles represent carbon atoms and filled circles radio-labelled carbon atoms.

Supplementary Figure 5

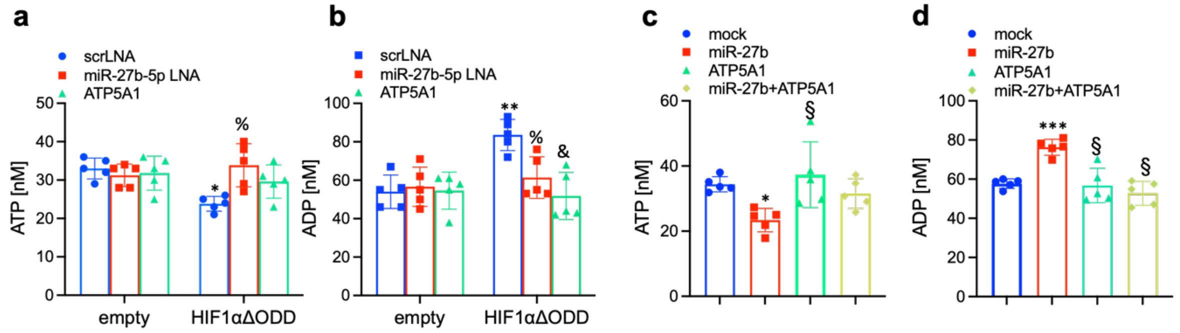

Supplementary Fig. 5 │ HIF1α regulates F_1F_0 ATP synthase activity via miR-27b
a-d, Quantification of ATP (**a, c**) and ADP (**b, d**) levels in NRCs transduced and treated as indicated. (n=5 biological replicates per group; results shown are the mean ± SD; *** $P < 0.001$, ** $P < 0.01$, * $P < 0.05$, vs. empty + scrLNA/mock, % $P < 0.05$ vs. scrLNA + HIF1αΔODD, & $P < 0.001$ vs. scrLNA + HIF1αΔODD; § $P < 0.01$ vs. ATP5A1; Two-way ANOVA followed by Tukey's post-test (**a, b**); One-way ANOVA followed by Tukey's post-test (**c , d**).

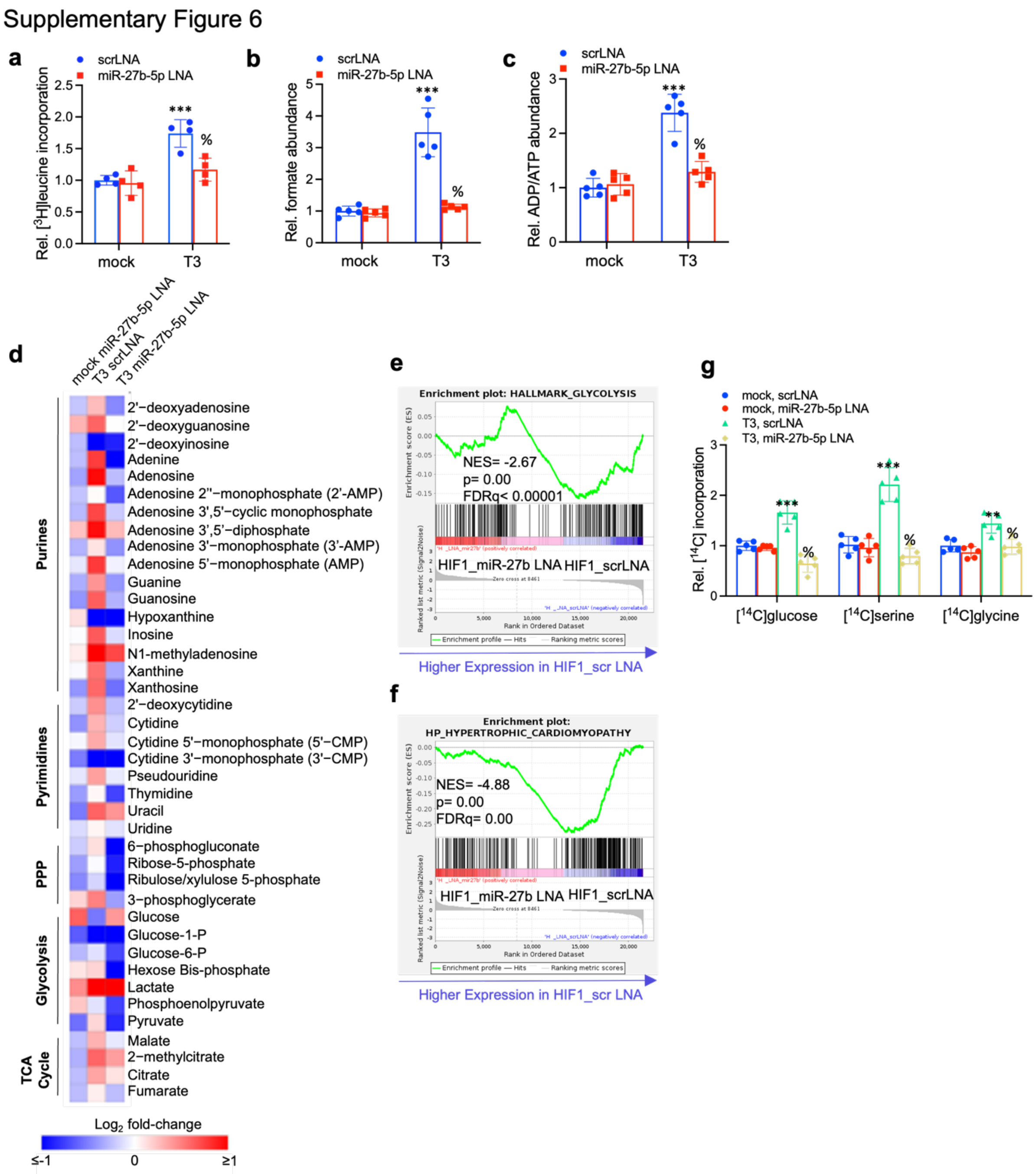

Supplementary Fig. 6 | T3 promotes purine biosynthesis and cell growth in a miR-27b-dependent manner

a, Evaluation of [^{3}H]leucine incorporation in NRCs stimulated with T3 and treated with scrLNA or miR-27b-5p LNAs. Data is represented as incorporated radioactivity relative to control (mock) NRCs treated with scrLNAs (set as 1.0). (n=4 biological replicates per group). **b,** Relative amount of formate in NRCs stimulated with T3 and treated with scrLNA or miR-27b-5p LNAs. (n=5 biological replicates per group). **c,** Relative ADP/ATP ratio in NRCs stimulated with T3 and

treated with scrLNA or miR-27b-5p LNAs. (n=5 biological replicates per group). *** $P < 0.001$ vs. mock + scrLNA, % $P < 0.01$ vs. T3 + scrLNA; Two-way ANOVA followed by Tukey's post-test. **d**, Heat map of relative metabolite abundance in NRCs stimulated with T3 and treated with scrLNA or miR-27b-5p LNAs. Depicted are metabolites with $\log_2$(fold change)>0.4 and adjusted *P* value<0.01 in at least one treatment group compared to corresponding control (n=4 biological replicates per group). **e, f** Enrichment plots of Gene set enrichment analyses for glycolysis (**e**) and hypetrophic cardiomyopathy (**f**) in NRCs transduced and treated as indicated. (n=3 biological replicates per group). **g,** Relative amount of [^{14}C]carbon derived from [^{14}C]glucose, [^{14}C]serine and [^{14}C]glycine incorporated into nucleic acids in NRCs stimulated with T3 and treated with scrLNA or miR-27b-5p LNAs. (n=5 biological replicates per group). Results shown are the mean ± SD; *** $P < 0.001$, ** $P < 0.01$ vs. mock + scrLNA, % $P < 0.05$ vs. T3 + scrLNA; Two-way ANOVA followed by Tukey's post-test.

Supplementary Figure 7

a

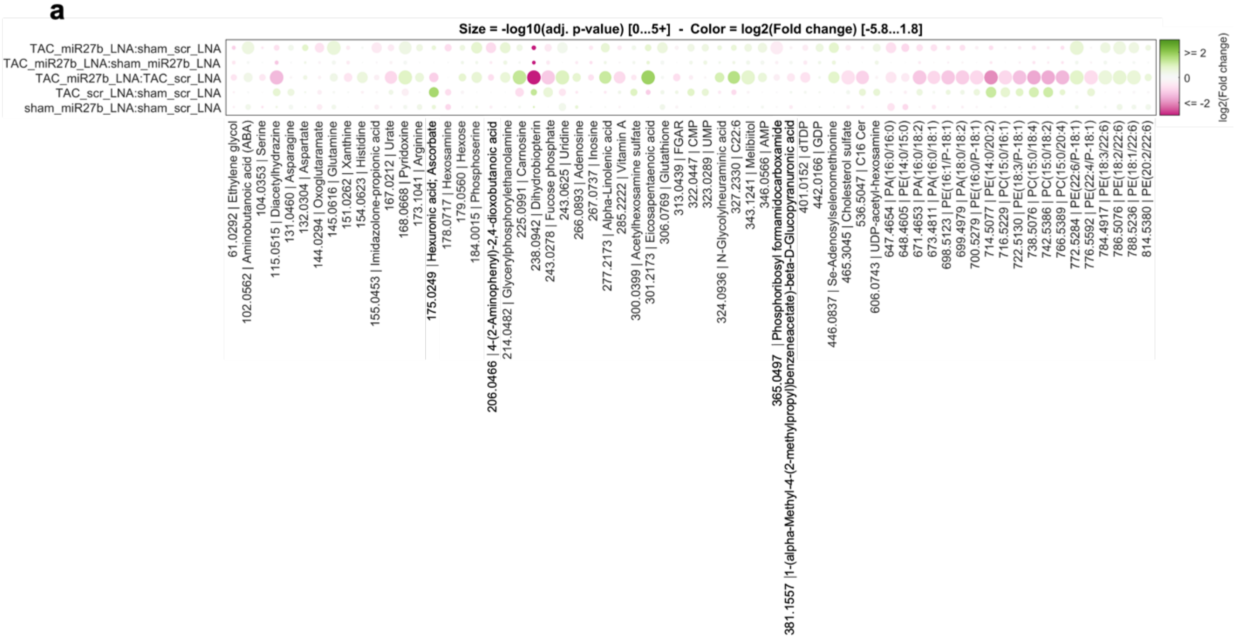

b

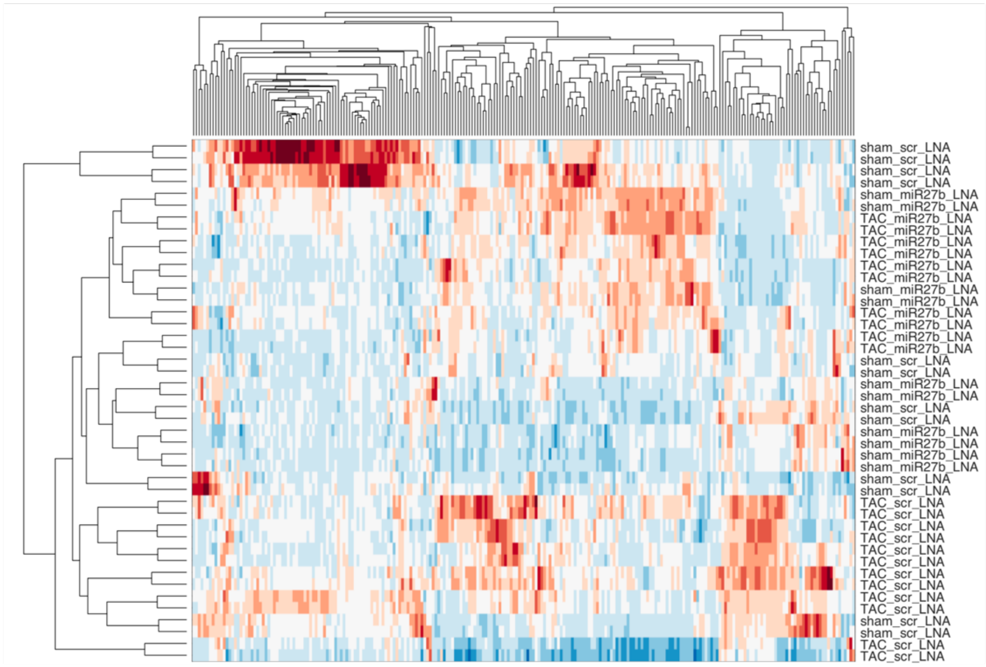

Supplementary Fig. 7 | Depletion of miR-27b decreases purine biosynthesis and reduces pressure-overload induced metabolic remodeling

a, Differential analysis of metabolites in left ventricles of sham or TAC-operated C57BL/6J mice injected with scrLNA or miR-27b-5p LNA; circle seize reflects $-\log_{10}$ (adj. p-value); circle color reflects $\log_2$ fold change compared to denoted control (n=6 biological replicates per group). **b,** Hierarchical cluster analysis of metabolites in denoted samples measured in duplicates (n=6 biological replicates per group).

Supplementary Figure 8

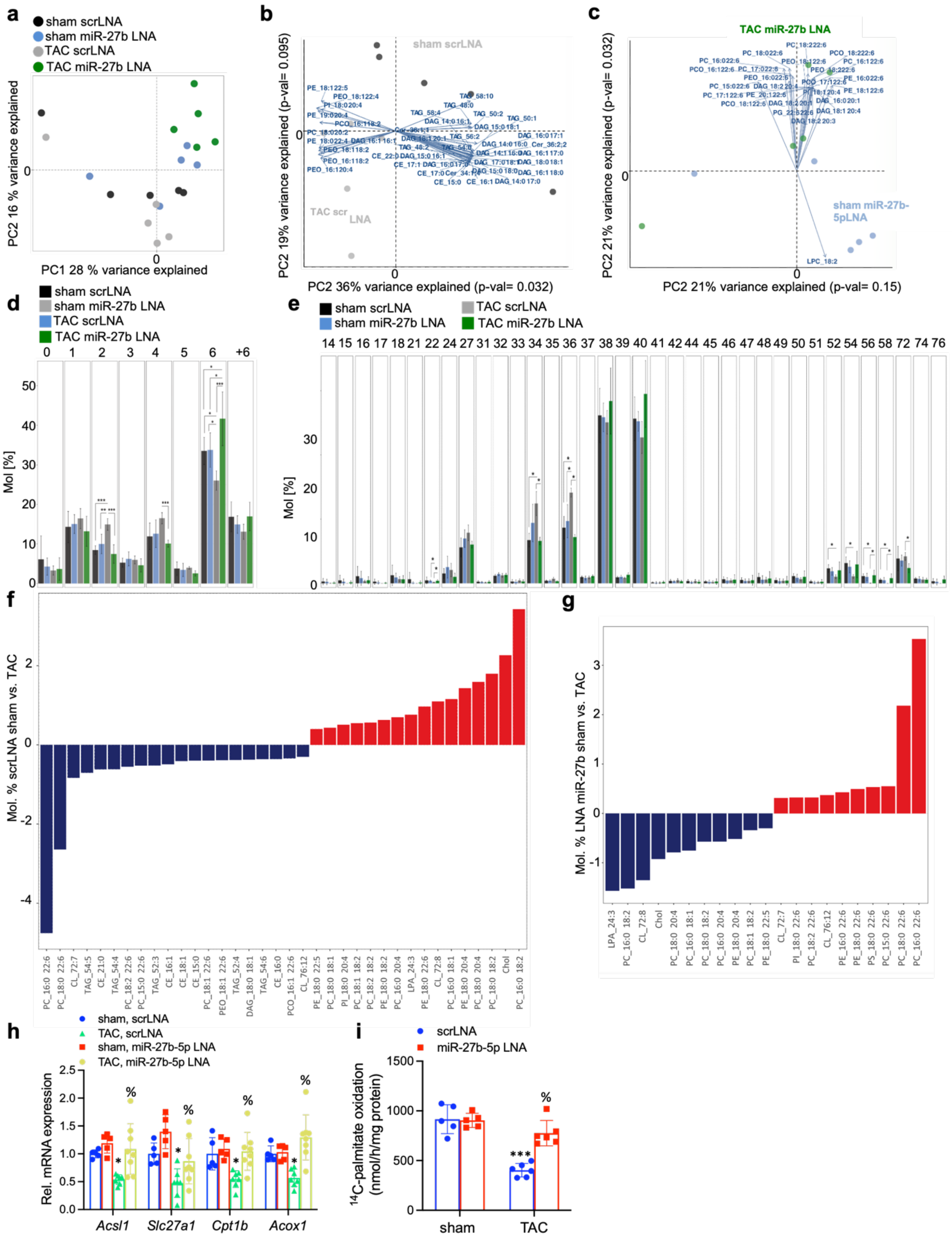

Supplementary Fig. 8 | miR-27b inhibits fatty acid oxidation in mice

a, Principal Component Analysis (PCA) of denoted cohorts. **b**, **c**, Principal Component Analysis performed separately on left ventricular samples of sham or TAC treated mice with active (scrambled, **b**) or LNA-mediated repressed miR-27b-5p (**c**). Arrows represent lipid species that concur to explain 15% of the variance encompassed by PC1 (Wilcoxon non-parametric signed-rank test; n= 5 per group). **d,** Barplot of the saturation profiles in the denoted four cohorts (mean +/- SD). Lipids were grouped according to the number of double bonds (db), and all species exceeding 6 db were gathered in a single group (6+). Analyzed lipids include the following 19 lipid classes cholesteryl esters (CE), ceramides (Cer), cholesterols (Chol), cardiolipins (CL), diacylglycerols (DAG), lyso-phosphatides (LPA), lyso-phosphatidylcholines (LPC), Lyso-phosphatidylethanolamines (LPE), ether linked LPE (LPE-O^-), lyso-phosphatidylinositols (LPI), phosphatidylcholines (PC), ether linked PC (PC-O^-), phosphatidylethanolamines (PE), ether linked PE (PE-O^-), phosphatidylglycerols (PG), phosphatidylinositols (PI), phosphatidylserines (PS), sphingomyelins (SM) and triacylglycerols. (adj. *P*-value< 0.001***, adj. *P*-value< 0.01**, adj. *P*-value< 0.1*; Wilcoxon non-parametric signed-rank test; n= 5 biological samples per group). **e,** Barplot of the total length profiles in the four denoted cohorts (mean +/- SD). Lipids (as analyzed in **d**) were grouped according to the total length of their acyl chains, i.e. number of carbon atoms. Significant comparisons are indicated with asterisks (adj. *P*-value< 0.001***, adj. *P*-value< 0.01**, adj. *P*-value< 0.1*; Wilcoxon non-parametric signed-rank test; n= 5 biological samples per group). **f,** Mean mol% abundance of lipid species in left ventricular biopsies of scrLNA treated TAC-operated mice *minus* mean mol% abundance of lipid species in sham-controls with. **g,** Mean mol% abundance of lipid species in TAC-operated mice injected with miR-27b-5p LNA *minus* mean mol% abundance of lipid species in sham-controls with repressed (LNA) miR-27b-5p. **h,** Relative expression of indicated mRNAs in C57BL/6J mice treated with scrLNA or miR-27b-5p LNAs and subjected to either sham or TAC surgery by qPCR. Data is normalized to sham-operated mice treated with scrLNA (set as 1.0). (n=5 for sham scrLNA, n=5 for sham miR-27b-5p LNA, n=7 for TAC scrLNA, n=8 for TAC miR27b-5p LNA; shown is mean ± SD; * $P < 0.05$ vs. sham scrLNA, % $P < 0.05$ vs. TAC scrLNA; Two-way ANOVA and Tukey's post-test). **i,** Oxidation of [1-^{14}C]-palmitate to $^{14}CO_2$ was measured in left ventricular biopsies from C57BL/6J mice subjected to sham or TAC surgery and treated with either scrLNA or miR-27b-5p LNAs. All values were normalised to the protein content. n=5 for sham scrLNA, n=5 for sham miR-27b-5p LNA, n=6 for TAC scrLNA, n=6 for TAC miR-27b-5p LNA; shown is mean ± SD; *** $P < 0.001$ vs. sham scrLNA, % $P < 0.05$ vs. TAC scrLNA ; Two-way ANOVA and and Tukey's post-test.

Supplementary Figure 9

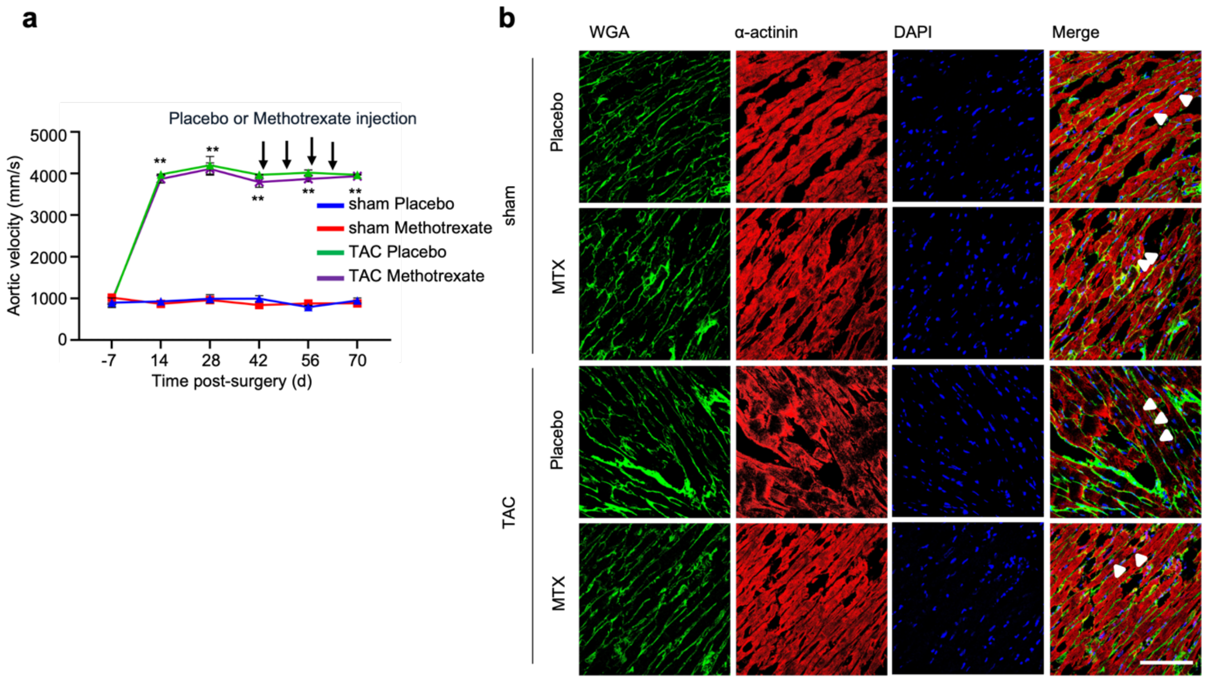

Supplementary Fig. 9 | Methotrexate inhibits miR-27b function in pathology

a, Aortic velocity across ligature, of sham and TAC mice administered the Placebo or MTX, respectively. n=4 for sham Placebo, n=5 for sham MTX, n=5 for TAC Placebo, n=6 for TAC MTX; shown is mean ± SEM; ** $P < 0.01$ vs. sham Placebo; two-tailed unpaired t-test. **b**, Representative images of WGA (green), α-actinin (red) and DAPI (blue) from sham and TAC mice treated with the Placebo or Methotrexate. Arrows indicate nucleation of cardiomyocytes. Scale bar is 100 µm. MTX, Methotrexate; WGA, Wheat germ agglutinin.

Supplementary Table 1

	AAV9-loxP-STOPcassette-loxP-miR-27b	
	Mlc2v-cre-	*Mlc2v-cre+*
n	6	8
Heart rate (bpm)	494.81 ± 22.74	446.51 ± 11.06
IVS;d (mm)	0.71 ± 0.02	0.73 ± 0.01
IVS;s (mm)	1.00 ± 0.03	0.98 ± 0.01
LVID;d (mm)	3.56 ± 0.04	4.00 ± 0.08*
LVID;s (mm)	2.33 ± 0.06	2.90 ± 0.07*
LVPW;d (mm)	0.71 ± 0.02	0.71 ± 0.01
LVPW;s (mm)	1.05 ± 0.02	0.95 ± 0.01
LVID Trace (CO) (ml/min)	17.17 ± 1.43	17.41 ± 0.85
LVID Trace (SV) (μl)	34.33 ± 1.40	38.99 ± 1.55
%FS	34.54 ± 1.30	27.58 ± 0.62*
%EF	64.49 ± 1.72	52.59 ± 1.01*
LV Mass (mg)	82.89 ± 2.64	104.15 ± 3.83*
LV Vol;d (μl)	53.10 ± 1.49	72.25 ± 3.32**
LV Vol;s (μl)	18.90 ± 1.21	34.39 ± 2.00**
LVW/BW (echo)	2.75 ± 0.05	3.18 ± 0.12 *
HW/BW (post sacrifice)	3.59 ± 0.07	3.98 ± 0.13*

Supplementary Table 1 | Echocardiographic analysis of Mlc2v-cre-/-cre+ mice injected with AAV9-loxP-STOPcassette-loxP-miR-27b viruses

IVS, intraventricular septum thickness at diastole (d) and systole (s); LVID, left ventricular internal diameter at diastole (d) and systole (s); LVPW, left ventricular posterior wall thickness at diastole (d) and systole (s); FS, fractional shortening; EF, ejection fraction; LVW/BW, left ventricular weight/body weight; HW/BW, heart weight/body weight. Values shown are mean ± s.e.m.; *P<0.05; **P<0.01; two-tailed unpaired t-test.

Supplementary Table 2

	scrLNA sham				
days post surgery	0	14	28	42	63
n	5	5	5	5	5
LVID;d (mm)	3.78 ± 0.13	3.53 ± 0.06	3.43 ± 0.12	3.28 ± 0.08	3.37 ± 0.07
LVID;s (mm)	2.62 ± 0.16	2.24 ± 0.09	1.99 ± 0.19	1.88 ± 0.10	1.98 ± 0.11
LVPW;d (mm)	0.72 ± 0.02	0.75 ± 0.03	0.74 ± 0.02	0.76 ± 0.01	0.78 ± 0.03
LVPW;s (mm)	0.98 ± 0.03	1.08 ± 0.05	1.13 ± 0.05	1.12 ± 0.05	1.16 ± 0.05
%FS	30.73 ± 2.20	36.68 ± 1.70	42.28 ± 3.57	42.68 ± 2.44	41.53 ± 2.39
%EF	58.78 ± 3.25	65.22 ± 0.32	73.64 ± 4.59	74.63 ± 2.68	73.3 ± 2.68
LVW/BW echo	3.46 ± 0.18	3.42 ± 0.11	3.26 ± 0.20	2.79 ± 0.16	2.88 ± 0.11
Aortic velocity (mm/sec)	848 ± 71	945 ± 56	1008 ± 87	939 ± 102	955 ± 36
HW/BW (post sacrifice)	3.98 ± 0.03				

	miR-27b LNA sham				
days post surgery	0	14	28	42	63
n	5	5	5	5	5
LVID;d (mm)	3.66 ± 0.27	3.40 ± 0.06	3.38 ± 0.13	3.28 ± 0.10	3.54 ± 0.12
LVID;s (mm)	2.53 ± 0.27	2.05 ± 0.05	1.99 ± 0.12	1.93 ± 0.07	2.18 ± 0.09
LVPW;d (mm)	0.70 ± 0.02	0.76 ± 0.02	0.78 ± 0.01	0.79 ± 0.00	0.79 ± 0.03
LVPW;s (mm)	0.97 ± 0.04	1.12 ± 0.04	1.20 ± 0.02	1.18 ± 0.02	1.11 ± 0.03
%FS	1.50 ± 4.15	39.68 ± 1.54	[illegible]1.30 ± 2.01	41.12 ± 0.79	38.31 ± 1.58
%EF	9.31 ± 5.93	71.25 ± 1.81	[illegible]3.09 ± 2.36	73.14 ± 0.96	69.39 ± 1.95
LVW/BW echo	3.45 ± 0.18	3.20 ± 0.16	3.15 ± 0.18	3.12 ± 0.16	3.28 ± 0.21
Aortic velocity (mm/sec)	799 ± 19	864 ± 84	802 ± 42	960 ± 45	876 ± 71
HW/BW (post sacrifice)	4.20 ± 0.18				

	scrLNA TAC				
days post surgery	0	14	28	42	63
n	7	7	7	7	7
LVID;d (mm)	3.79 ± 0.07	3.53 ± 0.09	3.72 ± 0.12	4.01 ± 0.15*	4.24 ± 0.18***
LVID;s (mm)	2.80 ± 0.08	2.54 ± 0.10	2.71 ± 0.12	3.33 ±0.15*	4.21 ± 0.25**
LVPW;d (mm)	0.71 ± 0.02	0.97 ± 0.04*	1.01 ± 0.04*	1.05 ± 0.05**	1.13 ± 0.07**
LVPW;s (mm)	0.92 ± 0.02	1.30 ± 0.05**	1.36 ± 0.06	1.29 ± 0.04	1.38 ± 0.05
%FS	28.60 ± 1.30	25.78 ± 0.33	27.09 ± 2.01	19.29 ± 1.25	17.17 ± 2.12**
%EF	55.90 ± 2.10	51.45 ± 3.18	53.23 ± 3.19	37.32 ± 2.95	36.02 ± 4.13**
LVW/BW echo	3.71 ± 0.18	4.80 ± 0.16*	5.27 ± 0.22*	5.74 ± 0.45**	7.37 ± 0.95***
Aortic velocity (mm/sec)	699 ± 29	4590 ± 182***	4612 ± 183***	4697 ± 132***	4964 ± 357***
HW/BW (post sacrifice)	7.02 ± 0.47				

	miR-27b LNA TAC				
days post surgery	0	14	28	42	63
n	8	8	8	8	8
LVID;d (mm)	3.93 ± 0.07	2.98 ± 0.14*	3.59 ± 0.17	3.91 ± 0.21	3.83 ± 0.25
LVID;s (mm)	2.76 ± 0.08	2.70 ± 0.20	2.57 ± 0.22	3.05 ± 0.33	2.96 ± 0.44
LVPW;d (mm)	0.72 ± 0.01	1.07 ± 0.04**	0.98 ± 0.04	1.02 ± 0.06*	0.93 ± 0.04*
LVPW;s (mm)	0.97 ± 0.02	1.47 ± 0.05**	1.32 ± 0.06*	1.25 ± 0.04	1.29 ± 0.05*
%FS	29.26 ± 1.64	27.38 ± 2.40	28.83 ± 2.96	15.28 ± 0.97*	27.52 ± 1.81
%EF	56.56 ± 2.53	52.37 ± 4.70	55.92 ± 4.58	32.62 ± 2.49**	54.79 ± 2.28
LVW/BW echo	3.85 ± 0.20	4.22 ± 0.16	4.98 ± 0.29*	5.73 ± 0.65*	5.62 ± 0.65*
Aortic velocity (mm/sec)	775 ± 40	4638 ± 78***	4301 ± 259***	4544 ± 364***	4843 ± 234***

HW/BW (post sacrifice) 6.06 ± 0.21

LVID, left ventricular internal diameter at diastole (d) and systole (s); LVPW, left ventricular posterior wall thickness at diastole (d) and systole (s); FS, fractional shortening; EF, ejection fraction; LVW/BW, left ventricular weight/body weight; HW/BW, heart weight/body weight. Values shown are mean ± s.e.m.; *P<0.05; **P<0.01; TAC scrLNA vs. sham scrLNA; miR-27b-5p LNA sham vs. miR-27b-5p LNA TAC; two-tailed unpaired t-test.

Supplementary Table 2 │ Echocardiographic analysis of sham- or TAC operated mice injected with scrLNA or miR-27b-5p LNAs

Supplementary Table 3

Gene	Forward Primer (5'-3')	Reverse Primer (5'-3')
Acox1	TAACTTCCTCACTCGAAGCCA	AGTTCCATGACCCATCTCTGTC
Acsl1	TGCCAGAGCTGATTGACATTC	GGCATACCAGAAGGTGGTGAG
Aopep		
Atp5a1	TCTCCATGCCTCTAACACTCG	CCAGGTCAACAGACGTGTCAG
Col1a1	CGATGGATTCCCGTTCGAGT	CGATCTCGTTGGATCCCTGG
Col3a1	CTGTAACATGGAAACTGGGGAAA	CCATAGCTGAACTGAAAACCACC
Cpt1b	GCACACCAGGCAGTAGCTTT	CAGGAGTTGATTCCAGACAGGTA
Hif1α	TGCTCATCAGTTGCCACTTC	CGGCATCCAGAAGTTTTCTC
Hprt1	TCAGTCAACGGGGGACATAAA	GGGGCTGTACTGCTTAACCAG
Ldha	TGTCTCCAGCAAAGACTACTGT	GACTGTACTTGACAATGTTGGGA
Nppa	AGATGAGGTCATGCCC	AAGCTGTTGCAGCCTA
Nppb	CCAGTCTCCAGAGCAATTCAAGAT	GCTAATTCACAAAGGACTCGAGGT
pre-mir27b	TGCAGAGCTTAGCTGATTGG	CCTTCTCTTCAGGTGCAGAAC
Slc2a1	CAGTTCGGCTATAACACTGGTG	GCCCCCGACAGAGAAGATG
Slc27a1	CGCTTTCTGCGTATCGTCTG	GATGCACGGGATCGTGTCT
TGFb1	CTCCCGTGGCTTCTAGTGC	GCCTTAGTTTGGACAGGATCTG
Vegfa	GCACATAGAGAGAATGAGCTTCC	CTCCGCTCTGAACAAGGCT

Supplementary Table 3 | qRT-PCR primers

78 Kassiri, Z. *et al.* Combination of tumor necrosis factor-alpha ablation and matrix metalloproteinase inhibition prevents heart failure after pressure overload in tissue inhibitor of metalloproteinase-3 knock-out mice. *Circulation research* **97**, 380-390 (2005).

79 Troilo, A. *et al.* HIF1alpha deubiquitination by USP8 is essential for ciliogenesis in normoxia. *EMBO Rep* **15**, 77-85 (2014).

80 Elion, E. A., Marina, P. & Yu, L. Constructing recombinant DNA molecules by PCR. *Current protocols in molecular biology / edited by Frederick M. Ausubel ... [et al* **Chapter 3**, Unit 3 17 (2007).

81 Casonato, A. *et al.* A new L1446P mutation is responsible for impaired von Willebrand factor synthesis, structure, and function. *J Lab Clin Med* **144**, 254-259 (2004).

82 Krishnan, J. *et al.* Dietary obesity-associated Hif1alpha activation in adipocytes restricts fatty acid oxidation and energy expenditure via suppression of the Sirt2-NAD+ system. *Genes & development* **26**, 259-270 (2012).

83 Love, M. I., Huber, W. & Anders, S. Moderated estimation of fold change and dispersion for RNA-seq data with DESeq2. *Genome Biol* **15**, 550 (2014).

84 Musso, G. *et al.* Generating and evaluating a ranked candidate gene list for potential vertebrate heart field regulators. *Genom Data* **6**, 199-201 (2015).

85 Schneider, C. A., Rasband, W. S. & Eliceiri, K. W. NIH Image to ImageJ: 25 years of image analysis. *Nature methods* **9**, 671-675 (2012).

86 Fukuzawa, J. *et al.* Cardiotrophin-1 increases angiotensinogen mRNA in rat cardiac myocytes through STAT3 : an autocrine loop for hypertrophy. *Hypertension* **35**, 1191-1196 (2000).

87 Huynh, F. K., Green, M. F., Koves, T. R. & Hirschey, M. D. Measurement of fatty acid oxidation rates in animal tissues and cell lines. *Methods Enzymol* **542**, 391-405 (2014).

88 Fuhrer, T., Heer, D., Begemann, B. & Zamboni, N. High-throughput, accurate mass metabolome profiling of cellular extracts by flow injection-time-of-flight mass spectrometry. *Anal Chem* **83**, 7074-7080 (2011).

89 Cimen, I. *et al.* Prevention of atherosclerosis by bioactive palmitoleate through suppression of organelle stress and inflammasome activation. *Sci Transl Med* **8**, 358ra126 (2016).

90 Sampaio, J. L. *et al.* Membrane lipidome of an epithelial cell line. *Proc Natl Acad Sci U S A* **108**, 1903-1907 (2011).

91 Ejsing, C. S. *et al.* Global analysis of the yeast lipidome by quantitative shotgun mass spectrometry. *Proc Natl Acad Sci U S A* **106**, 2136-2141 (2009).

92 Surma, M. A. *et al.* An automated shotgun lipidomics platform for high throughput, comprehensive, and quantitative analysis of blood plasma intact lipids. *Eur J Lipid Sci Technol* **117**, 1540-1549 (2015).

93 Liebisch, G. *et al.* High throughput quantification of cholesterol and cholesteryl ester by electrospray ionization tandem mass spectrometry (ESI-MS/MS). *Biochim Biophys Acta* **1761**, 121-128 (2006).

94 Herzog, R. *et al.* LipidXplorer: a software for consensual cross-platform lipidomics. *PLoS One* **7**, e29851 (2012).

95 Herzog, R. *et al.* A novel informatics concept for high-throughput shotgun lipidomics based on the molecular fragmentation query language. *Genome Biol* **12**, R8 (2011).

96 R: A Language and Environment for Statistical Computing (R Foundation for Statistical Computing, Vienna, Austria, 2017).
97 factoextra: Extract and Visualize the Results of Multivariate Data Analyses (2017).
98 Wickham, H. Reshaping Data with the reshape Package. *Journal of Statistical Software* **21**, 1-20 (2007).
99 Wickham, H. *ggplot2: Elegant Graphics for Data Analysis*. (Springer-Verlag New York, 2009).
100 Dweep, H., Sticht, C., Pandey, P. & Gretz, N. miRWalk--database: prediction of possible miRNA binding sites by "walking" the genes of three genomes. *J Biomed Inform* **44**, 839-847 (2011).
